# PPR9 mediates mitochondrial *nad* transcript maturation required for complex I biogenesis and early plant development in Arabidopsis

**DOI:** 10.1007/s11103-026-01769-9

**Published:** 2026-10-21

**Authors:** Ella Kobaivanov, Tair Maltz, Marketa Kittel, Roei Matan, Ron Mizrahi, Noa Carmi, Oren Ostersetzer-Biran

**Affiliations:** 1 Department of Plant and Environmental Sciences, The Alexander Silberman Institute of Life Sciences, The Hebrew University of Jerusalem, Jerusalem, Israel; 2 Institute of Parasitology, Biology Centre, Czech Academy of Sciences, České Budějovice, Czech Republic

**Keywords:** Pentatricopeptide (PPR), RNA metabolism, Splicing, Group II introns, Mitochondria, Arabidopsis

## Abstract

The biogenesis and function of plant mitochondria relies on the expression of the organellar genome, which involves extensive post-transcriptional processing of organellar transcripts in order to generate mature functional RNAs. This includes the removal of introns from many essential organellar transcripts through splicing, a process mediated by diverse nuclear-encoded RNA-processing factors, including numerous members of the pentatricopeptide repeat (PPR) protein family. Here, we report the analysis of PPR9 (At1g03560), a mitochondrial P-class PPR protein originally identified in genetic screens for essential protein cofactors. Loss of PPR9 function results in embryonic arrest at early stages of seed development. In vitro rescued homozygous *ppr9* mutant plants could be recovered and maintained using a modified in vitro embryo-rescue method. The mutants display delayed growth and development which are associated with altered mitochondrial functions. Molecular analyses reveal that PPR9 functions in the splicing of several mitochondrial transcripts encoding respiratory complex I (CI) subunits, including *nad2* intron 3 and *nad7* introns 1 and 2. Accordingly, CI assembly is disrupted in *ppr9* plants, resulting in altered OXPHOS activity and broad physiological defects. Together, these findings identify PPR9 as a key regulator of mt-RNA maturation in Arabidopsis, enabling CI biogenesis and optimal respiratory functions, and further highlight the roles of nuclear-encoded factors in coordinating mitochondria functions and (early) plant growth and development.

**Supplementary Information:**

The online version contains supplementary material available at https://doi.org/10.1007/s11103-026-01769-9.

## Key message

Our study identifies PPR9 as a key mitochondrial RNA-processing factor required for the splicing of multiple group II introns that reside within genes encoding subunits essential for respiratory complex I biogenesis in Arabidopsis. By linking nuclear control of RNA maturation to mitochondrial function and plant development, this work emphasizes a critical layer of coordination between organellar gene expression, mitochondria function and developmental regulation in land plants.

**Supplementary Information:**

The online version contains supplementary material available at https://doi.org/10.1007/s11103-026-01769-9.

## Introduction

Mitochondria are essential organelles that serve as major sites of energy production, as well as central hubs for diverse metabolic and signaling pathways (Braun 2020; Møller et al. 2021). Mitochondria in plants contribute to key developmental processes, such as developmental regulation and stress responses. Reflecting their origin from an ancient bacterial ancestry, most mitochondria retain their own genome (mtDNA, mitogenome), with intrinsic bacterial-like transcriptional and translational machineries (Gualberto and Newton 2017). However, the vast majority of organellar proteins, as well as some tRNAs, are encoded by nuclear gene-loci and imported into the mitochondria (Braun 2020; Møller et al. 2021). The biogenesis of a fully functional oxidative phosphorylation (OXPHOS) machinery relies on the coordinated expression of both nuclear- and mitochondria-encoded proteins. These processes are essential for maintaining optimal respiratory functions and cellular homeostasis, thereby enabling optimal plant growth and development.

Although all mitochondria are thought to share a common ancestor, they have diverged considerably across different eukaryotic lineages. The mtDNA in plants differ markedly from their animal counterparts in both size and structural complexity. Whereas animal mitogenomes are typically compact (~ 17 kb) and show high conservation in genome structure and gene content, those of plants (especially land plants) vary in size, ranging from tens of kilobases to over 10 Mb, and are further characterized by multipartite and highly recombinogenic structures (Gualberto and Newton 2017). This genetic complexity is also evident in mitochondrial gene-expression. Plant mitochondrial transcripts must undergo extensive post-transcriptional processing, including end trimming, RNA editing, and splicing, to generate functional rRNAs, tRNAs and mRNAs (Hammani and Giege 2014; Small et al. 2023; Zmudjak and Ostersetzer-Biran 2018). These essential RNA processing steps enable the synthesis of mitochondria-encoded proteins, which essential for respiration and various other metabolic processes.

A noticeable feature of plant mitogenomes is the high abundance of intron sequences that reside in many protein-coding genes, particularly within transcripts encoding subunits of the NADH dehydrogenase enzyme (i.e., complex I, CI), and also in genes encoding cytochrome c oxidase complex (CIV) subunits, cytochrome c maturation (CCM) factors, ribosomal protein biogenesis, but may reside in other genes as well (Schmitz-Linneweber et al. 2015; Zmudjak and Ostersetzer-Biran 2018). The majority of the organellar introns in land plant mitochondria are identified as group II-type, an ancient group of catalytic ribozymes that are evolutionarily related to non-LTR retroelements and share common features with spliceosomal introns and telomerases (Lambowitz and Belfort 2015; Zhao and Pyle 2017; Zimmerly and Semper 2015). Canonical group II RNAs are characterized by a conserved secondary structure comprising of six domains (D1-D6), which allow the folding of an active tertiary conformation that undergoes two sequential self-transesterification reactions required for splicing (Zhao and Pyle 2017; Zimmerly and Semper 2015). Yet, for their efficient excision in vivo, these introns rely on their own intron-encoded maturase proteins (IEPs, or MATs). The model introns in this class may also assisted by additional factors, such as RNA helicases, which like the cognate MAT factors, most likely assist in stabilizing the intron structure and thus promoting efficient RNA catalysis (Halls et al. 2007; Mizrahi and Ostersetzer-Biran 2024; Mizrahi et al. 2022; Russell et al. 2013; Zhao and Pyle 2017). Throughout evolution, the mitochondrial group II introns in plants have diverged extensively in sequence and have often lost their cognate MAT proteins (Mizrahi et al. 2022). In some cases, the introns have further fragmented into independently transcribed gene fragments that assemble *in trans* to facilitate exon joining (Mizrahi et al. 2022). The sequence, and likely structural, alterations of the organellar introns were accompanied by the recruitment of nuclear-encoded cofactors, to facilitate the splicing of the non-canonical introns in plant mitochondria, and are likely further contributing to increased regulatory complexity of organellar gene expression (Best et al. 2020). Plant organellar splicing factors comprise a diverse set of RNA-binding proteins, including MAT-related proteins encoded by stand-alone nuclear genes (nMATs) and RNA helicases, as well as various other factors such as CRM-domain proteins, mTERFs, PORR-domain proteins, and members of the pentatricopeptide repeat (PPR) protein family, which has greatly expanded in land plants and is the focus of this study (Small et al. 2023; Zmudjak and Ostersetzer-Biran 2018).

The PPR proteins constitute one of the largest protein families in angiosperms, which are characterized by tandem repeats of a degenerate 35-amino acid motif (with a helix-turn-helix structure) (Small and Peeters 2000), enabling a modular and sequence-specific recognition of single-stranded RNA, where each PPR motif is postulated to bind a specific ribonucleotide (see e.g., (Barkan and Small 2014; Gully et al. 2015; Shen et al. 2015). Many of the plant PPR proteins contain putative mitochondria localization signals, where they are expected to regulate diverse aspects of organellar RNA metabolism, including RNA processing and trimming, transcript stabilization, RNA editing (primarily C-to-U deamination), and the splicing of transcripts containing group II introns (Gutmann et al. 2020; Small et al. 2023; Zmudjak and Ostersetzer-Biran 2018). Recent studies suggest that PPR proteins may also participate in the regulation of organellar translation (Grüttner et al. 2022; Rugen et al. 2024; Waltz et al. 2019).

Despite substantial advances in understanding the RNA-recognition code and target specificity of PPR factors (Barkan and Small 2014; Cheng et al. 2016; Gutmann et al. 2020; McDermott et al. 2019; Rojas et al. 2019), as well as their specific roles in RNA editing (Small et al. 2020), the molecular mechanisms by which PPR proteins facilitate splicing remain less understood. Here, we report the functional characterization of PPR9 (At1g03560), a P-class PPR protein that is essential for early embryogenesis in *Arabidopsis thaliana*. Experimental analyses demonstrate that PPR9 is required for the excision of three mitochondrial group II intron: *nad2* intron 3 and *nad7* introns 1 and 2. Defects in splicing of these introns in *ppr9* mutant-plants impairs holo-CI biogenesis, leading to notable physiological defects. The effects of PPR9 disruption on organellar biogenesis and the physiology of embryo-rescued Arabidopsis mutant-lines are described in detail.

## Results

### The *PPR9* (At1g03560) gene-locus encodes a P-type PPR protein predicted to localize to mitochondria

**Fig. 1:**
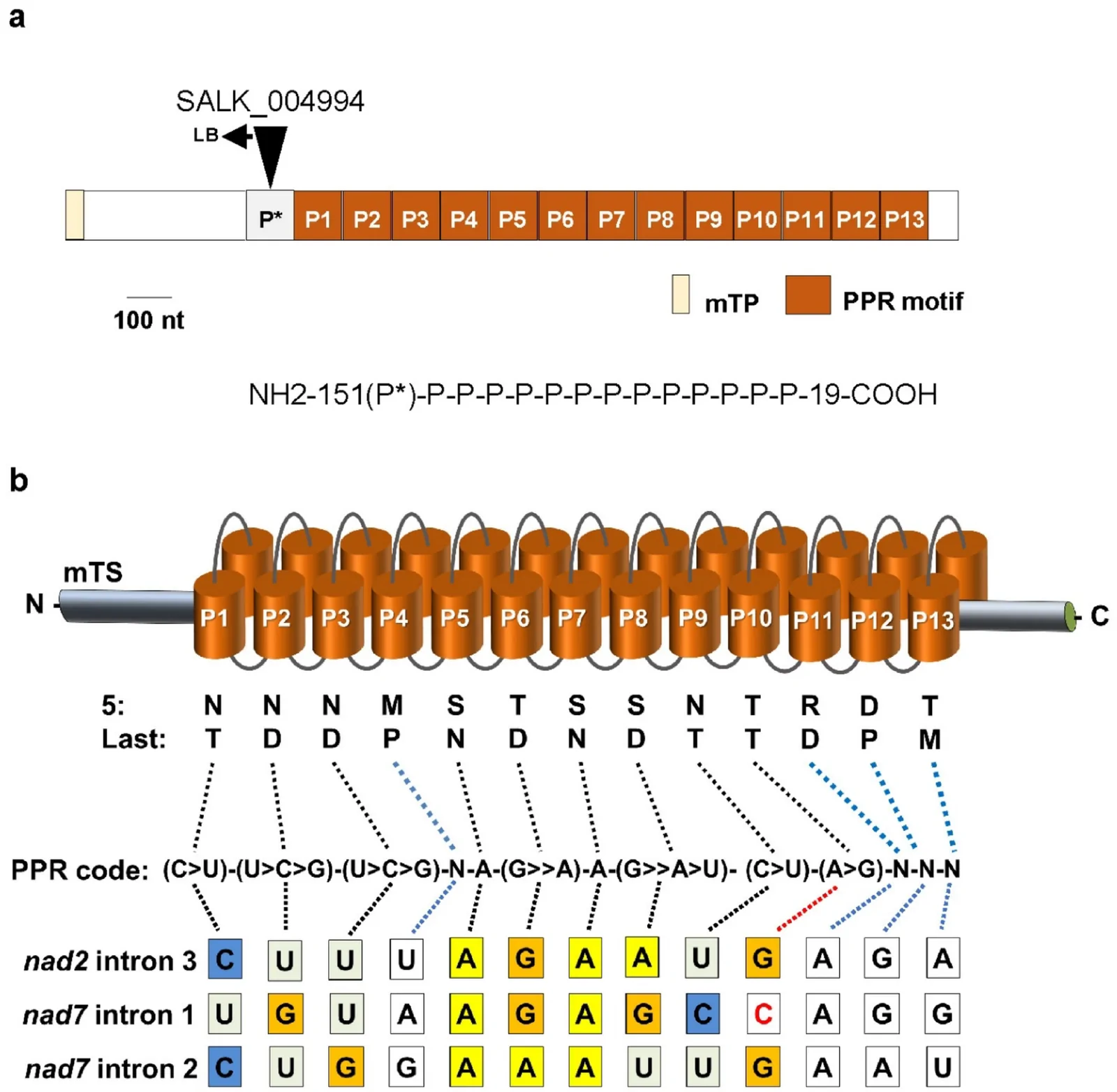
The *AT1G03560* gene-locus (*PPR9*) encodes a putative mitochondria-localized PPR protein. a Schematic representation of the *PPR9* (*AT1G03560*, UniProt Q9LR67) gene structure. In silico analysis suggests the presence of 13 conserved P-class PPR motifs (P1-P13), as well as an additional putative PPR motif (labeled as P*; amino acids 152–186). Numbers correspond to amino acid residues not assigned to any defined motif. The predicted mitochondrial targeting sequence (i.e., mTP, mitochondrial transit peptide) and the T-DNA insertion site in the SALK_004994 line (*ppr9-1*) within the PPR9 coding region is indicated above the gene structure figure. **b** Prediction of putative RNA-binding sites of PPR9. The fifth and last amino acids of each PPR repeat are indicated below the corresponding motifs. The predicted RNA-recognition code is shown beneath the figure. ‘N’ denotes PPR motifs with unassigned nucleotide specificity. Postulated RNA-binding sites within *nad2* intron 3 and *nad7* introns 1 and 2 (Table S1) are illustrated, with boxes color-coded as follows: U (light green), C (blue), G (orange), A (yellow), and unassigned bases (white). Red color indicates mismatches between the predicted PPR code and target nucleotides

During the evolution of land plants, the mitogenomes have acquired remarkable structural plasticity, with extensive variation in genome size, architecture and expression patterns (especially at the RNA level) among species (Best et al. 2020; Broz et al. 2025; Gualberto and Newton 2017; Knoop 2012). To gain insights into the mechanisms of mt-RNA processing steps in plants, we screened a collection of publicly available *A. thaliana* T-DNA insertional mutants (i.e., The Arabidopsis Information Resource, TAIR, database) that reside in genes that encode putative mitochondria-targeted P-type PPR proteins. Among these, was a heterozygous mutant-line carrying a T-DNA insertion (SALK_004994) in the *AT1G03560* gene-locus (UniProt Q9LR67, or PPR9_ARATH). The deduced product of the *PPR9* gene-locus encodes 660 amino acids, and harbors at least 13 identified PPR (P) motifs with a predicted topology of NH_2_-151-(P*)-P-P-P-P-P-P-P-P-P-P-P-P-P-19-COOH (Fig. 1a, Supplementary Fig. S1a). ‘P’ denotes canonical PPR motifs (Cheng et al. 2016; Yan et al. 2019), whereas the ‘P* region (amino acids 152–186) (Figs. 1a and S1a) corresponds to a non-canonical PPR-like motif, as predicted by the PPR-code (Cheng et al. 2016) and the AlphaFold structural modeling server (Fig. S1b). Numbers indicate the lengths (in amino acid residues) of N- and C-terminal regions that do not correspond to any recognized protein motifs or conserved domains. Notably, the P* region is diverged across land plants. Some homologs, including those from *Arabidopsis lyrata* (NP_171855.1), *Brassica napus* (BNAA10G02000D), *Brassica rapa* (BRA033410), *Solanum lycopersicum* (Solyc10g008510.1), and *Thellungiella parvula* (TP1G02490), contain only 13 PPR motifs, with the corresponding P* region is no longer recognized as a canonical PPR repeat (Cheng et al. 2016), whereas homologs from *Vitis vinifera* (GSVIVT01030557001) or *Ricinus communis* (27965.M000110) may harbor 14 PPR motifs, according to PPR code predictions (Cheng et al. 2016; Jumper et al. 2021).

TargetP (Emanuelsson et al. 2000), UniProt (Wu et al. 2006) and SUBA4 (Hooper et al. 2017) further predict the presence of a 53-amino acid that share homology with specific mitochondrial targeting sequences (i.e., mTP, mitochondrial transit peptide), at the N-terminus of PPR9 (Figs. 1a and S1a). Likewise, the deduced amino acids sequences of the orthologous proteins in *(A) lyrata*, *(B) napus*, *B. rapa*, *S. lycopersicum*, *T. parvula*, *V. vinifera* and *R. communis* are all predicted to be localized to the mitochondria by the TargetP server. The AlphaFold-predicted structure of *A. thaliana* PPR9 indicates a canonical superhelical solenoid fold composed of 13 PPR motifs (Fig. S1b), with an internal positively charged groove consistent with the RNA-binding surfaces described for PPR proteins (Gully et al. 2015; Shen et al. 2016).

PPR proteins are characteristically defined as sequence-specific RNA-binding factors (Barkan and Small 2014; Gully et al. 2015; Shen et al. 2015; Small et al. 2020, 2023), in which the RNA recognition is based on a combinatorial code defined by the amino acids found at positions 5 and 35 (or the last residue in short and long PPR domains) of each motif, which together define the nucleotides binding site (Barkan et al. 2012; Cheng et al. 2016; Takenaka et al. 2013). Based on the RNA-binding code, some PPR proteins exhibit predicted binding sites that accurately reflect the specific RNA targets validated further through genetic analyses. In other cases, however, the predicted RNA-binding sites is less defined or insufficient to accurately indicate the native target region. When the PPR code was applied to the 13 identifiable motifs of PPR9 (Fig. 1b), it yielded a highly broad range of potential binding sites within the last updated mtDNA sequence of *A. thaliana* (Sloan et al. 2018). In total, 203 possible recognition sites are found in the mitogenome of *A. thaliana* (Best et al. 2020; Sloan et al. 2018). These are distributed across multiple gene regions (excluding non-coding/intergenic regions): 19 sites are located within 5′ or 3′ untranslated regions (UTRs), 51 sites within coding (exonic) sequences, and 54 sites are identified within intron regions (Table S1, ordered according to their relative matching scores). This ambiguity emphasizes the need for genetic and biochemical assays to elucidate the molecular function of PPR9, by establishing its intracellular location, the native RNA targets and roles in gene expression, in vivo.

#### *PPR9* encodes a lowly-expressed mitochondria-localized PPR protein

**Fig. 2:**
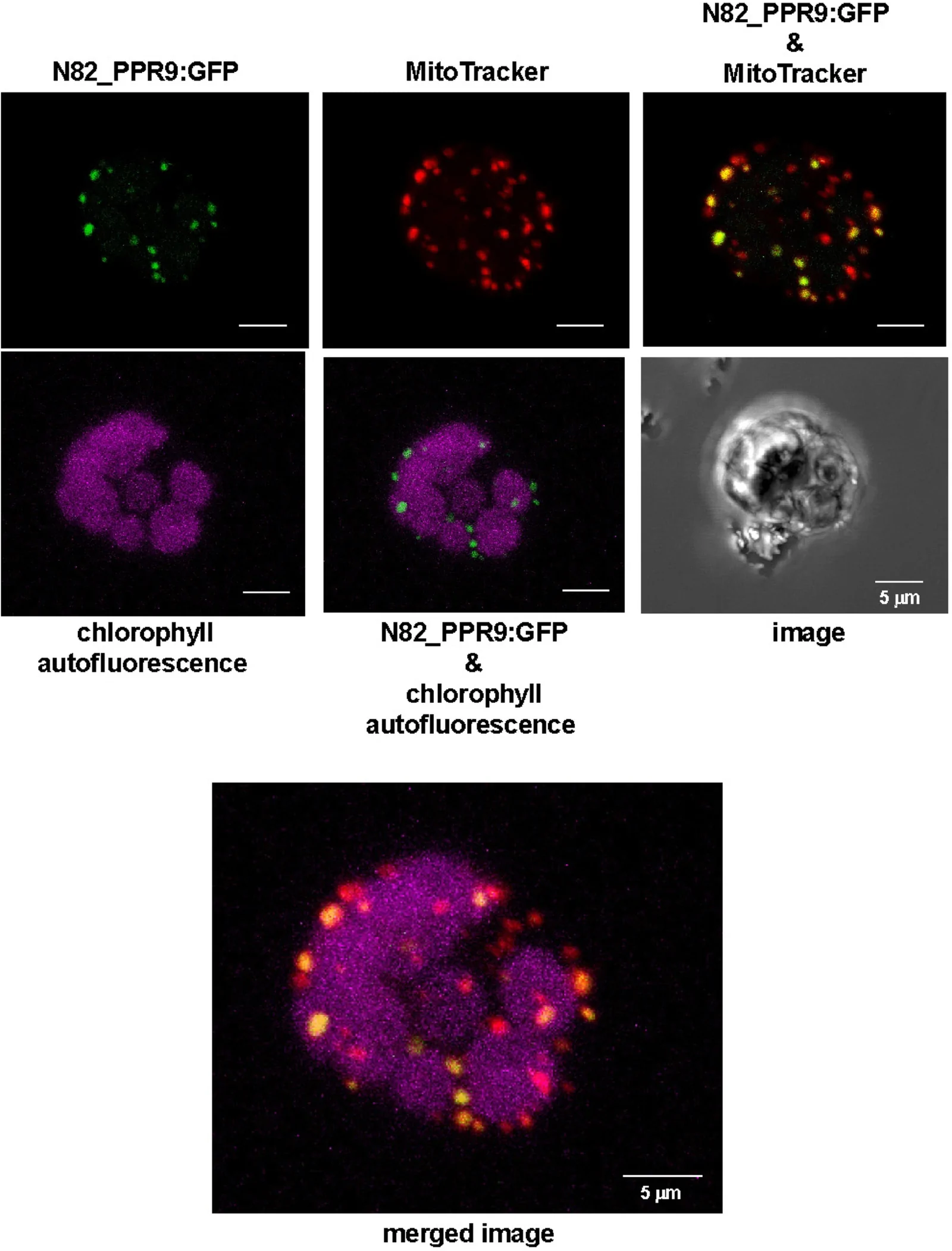
PPR9 is targeted to mitochondria, as revealed by subcellular localization analysis. Protoplasts were prepared from P. patens seedlings grown on sterile MS medium and transformed with the pCAMBIA-1302 vector containing a GFP sequence fused in-frame to the N-terminal region (246 bp) of the PPR9 (AT1G03560) coding sequence, under the control of CaMV 35 S promoter and a NOS terminator sequence at the 3′ end. The figure shows confocal N82_PPR9:GFP fluorescence (green, top left panel), MitoTracker (red, top middle panel) signals, GFP and MitoTracker overlay (top right panel), chlorophyll autofluorescence (purple, bottom left panel), GFP and chlorophyll autofluorescence (bottom middle panel), a bright-field image (grayscale, bottom right panel). The lower panel shows a merged (GFP, MitoTracker and chlorophyll autofluorescence) image. Scale bars represent 5.0 μm

Expression analysis using publicly available microarray and transcriptomic datasets (Huala et al. 2001; Luna-García et al. 2023) indicates that PPR9 displays differential expression across developmental stages, with elevated transcript levels in embryonic tissues, young developing leaves, the shoot apex, apical root regions, and flowers (Fig. S2). Here, we further analyzed the intracellular location of PPR9 protein, in vivo. For this purpose, we created a fusion protein construct containing the first 82 amino acids of PPR9 in-frame with eGFP (N82-PPR9:GFP), which was expressed in protoplasts obtained from *P. patens* (a system that allows an efficient protoplast preparation and provides reliable GFP localization assays applicable to angiosperm proteins (Thévenin et al. 2012, 2016). The confocal microscopy analyses showed that the N82_PPR9:GFP signals appear as globular-shaped particles that co-localized with the MitoTracker marker, a mitochondrion specific fluorescent probe (Fig. 2). The confocal microscopy did not reveal any additional intracellular localization, such as chloroplasts (as indicated by chlorophyll autofluorescence), the nucleus, or the cytoplasm (Fig. 2, merged image). These results strongly support the in silico predictions, as well as publicly available proteomic data (Fuchs et al. 2020) that showed that PPR9 is targeted to the mitochondria, where it is postulated to function in post-transcriptional regulation of organellar gene expression and/or RNA metabolism (Hammani and Giege 2014; Small et al. 2023; Zmudjak and Ostersetzer-Biran 2018). 

### PPR9 functions are required during early embryo development in *Arabidopsis thaliana*

**Fig. 3:**
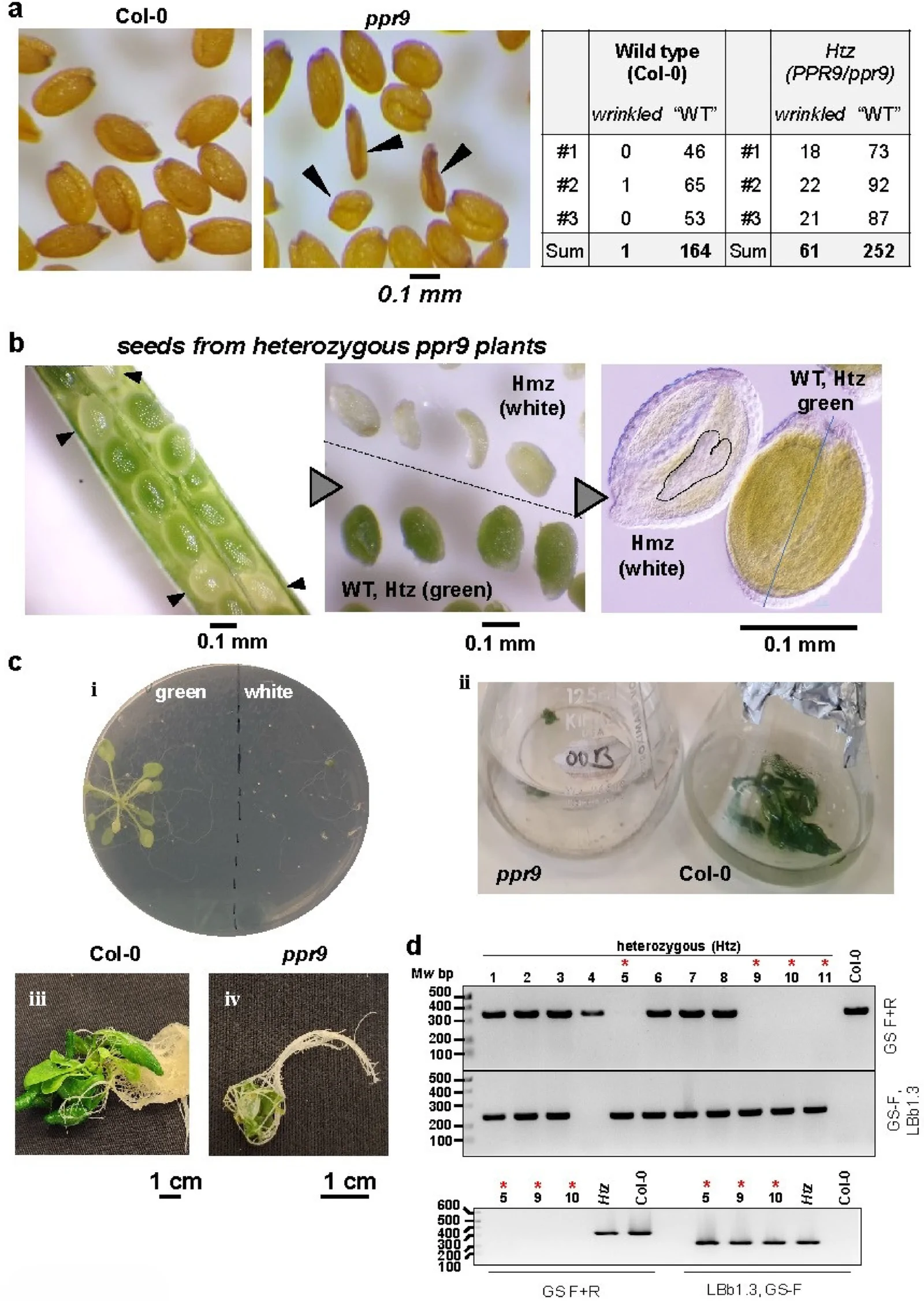
Loss of PPR9 causes abnormal seed development and embryonic arrest, which can be rescued by in vitro embryo-rescue methods. **a** Seeds collected from mature siliques of wild type (Col-0) and heterozygous *ppr9* plants. Arrows indicate abnormal *ppr9* mutant seeds, which appear shrunken and darker. **b** Representative images of green (wild type or heterozygous, Htz) and white (homozygous, Hmz, *ppr9*) seeds observed in mature green siliques of heterozygous *ppr9* plants (left panel). The morphology of green and white seeds collected from the same silique is shown in the middle panel. The right panel presents differential interference contrast (DIC; Nomarski) microscopy images of a green seed, containing a fully developed embryo, and a white seed harboring an embryo arrested at the torpedo-to-walking-stick stage. The arrested embryo is highlighted in the image. **c** Embryo-rescue procedure. Seeds collected under sterile conditions from surface-sterilized immature siliques of Col-0 or heterozygous *ppr9* plants were sown on MS-agar plates supplemented with 1% sucrose and vitamins (*i*), and grown under standard growth-chamber conditions (*ii*). In vitro rescued homozygous *ppr9* mutants (~ 2 month-old) exhibit abnormal morphology and developmental phenotypes, often displaying a bushy-like appearance (*iii*, *iv*), similar to that reported for other Arabidopsis mutants affected in mt-RNA metabolism (Best et al. 2023; Dahan et al. 2014; Shevtsov-Tal et al. 2021). **d** Genetic screening of seedlings obtained from the siliques of heterozygous plants. Seedlings grown on MS agar plates were genotyped using both gene-specific (GS; Table S3) and T-DNA-specific oligonucleotides (LBb1.3 forward and *ppr9* reverse; Table S3). The data show that the progeny of the heterozygous plants segregated into one wild type (line 4), heterozygous (lines 1–3 and 6–8), and in vitro rescued homozygous mutant lines (seedlings no. 5 and 9-to-11). Col-0 plants (i.e., line 12) were used as the wild type control. The lower panel shows PCR-based genetic analysis of the progeny derived from in vitro rescued *ppr9* mutants (*ppr9* lines 5, 9, and 10) obtained from seeds collected from heterozygous (*PPR9/ppr9*) parental plants, as well as heterozygous (Htz) and wild type (Col-0) plants

The characterization of PPR9 functions was initially studied by a reverse genetic approach. Analysis of the SIGnAL database identified a single T-DNA insertion line in the *PPR9* coding region, SALK_004994, with the T-DNA located 536 nucleotides downstream of the AUG translation initiation codon (Fig. 1a). Genetic screening revealed that under optimal growth conditions (see Materials and methods), no homozygous plants could be obtained from the progeny of the heterozygous *ppr9* line. Microscopic analysis showed that the vast majority (about 98%) of seeds in wild type siliques displayed a normal morphology, whereas about 24% (61 out of 252) of the seeds produced by heterozygous (*PPR9*/*ppr9*) plants were shrunken seeds (i.e., a ‘wrinkled’ seed morphology; (Pinfield-Wells et al. 2005) (Fig. 3a), consistent with the expected 1:3 segregation ratio of a recessive embryo-defective allele (χ^2^ = 0.085, *P* = 0.77). The abnormal seeds failed to germinate under standard growth conditions when sown in MS-agar plants or soil. The heterozygous *ppr9* plants exhibited no visible abnormalities (Fig. S3a), suggesting that *PPR9* encodes an essential factor, and that the complete loss of PPR9 functions result in embryonic developmental arrest or lethality. Microscopic (Nomarski, DIC) analysis of mature-green siliques (10–12 days post-anthesis, DPA) showed that green seeds contained typical fully developed embryos, whereas the white seeds of the same progeny harbored embryos that were arrested at the torpedo to walking-stick stages (Fig. 3b). These phenotypes coincide with various other Arabidopsis mutants affected in mitochondria biogenesis and function (Colas des Francs et al. 2014; Ostersetzer-Biran 2016).

Embryo rescue techniques have been successfully applied to recover several Arabidopsis mutants exhibiting germination-defective phenotypes (Franzmann et al. 1989). Equivalent approaches have been also used to rescue several mutants impaired in mitochondrial biogenesis or function in Arabidopsis, including *cod1* (Dahan et al. 2014), *ndufv1* (Kuhn et al. 2015) *cal1/cal2* (Cordoba et al. 2016, Fromm et al. 2016), *nmat3* (Shevtsov-Tal et al. 2021), *msp1* (Best et al. 2023) and *pcis1* (Kobaivanov et al. 2026). Following our own laboratory strategy (Shevtsov-Tal et al. 2021), we selected white and green seeds collected from green-mature siliques of heterozygous *ppr9* plants (10–12 DPA). These were plated on MS-agar plates supplemented with 1% sucrose and a vitamin mix (see Materials and methods), and transferred to a controlled growth chamber. Across three independent experiments, 7 of 16, 5 of 19, and 3 of 12 white seeds germinated on MS medium (Fig. 3c i). To obtain sufficient biomass for downstream analyses (e.g., RT-qPCR and BN-PAGE), in vitro rescued homozygous seedlings at the L6 developmental stage (Boyes et al. 2001) were transferred to liquid MS medium (Fig. 3c ii–iv) and cultured under the conditions specified in the ‘Materials and Methods’ section, with gentle agitation (50–100 rpm), essentially as previously described (Best et al. 2023; Shevtsov-Tal et al. 2021). Many of the *ppr9* seedlings died within 2 to 4 weeks after germination, as well as following transfer to liquid MS medium. The seedlings that survived on the media (Figs. 3c, S3b) were used for further RNA, protein and organellar activity analyses. PCR-based genotyping (Fig. 3d) confirmed that the seedlings regenerated from the white seeds (Fig. 3c iv) were indeed homozygous for the *ppr9* mutant allele, whereas seedlings derived from green seeds of the same siliques were either wild type or heterozygous for the SALK_004994 insertion (Fig. 3c and d). As previously noted for various embryo-defective (*emb*) mutants (Boyes et al. 2001), including those with defects in mitochondrial biogenesis (e.g., (Best et al. 2023; Colas des Francs et al. 2014; Dahan et al. 2014; Kuhn et al. 2015; Shevtsov-Tal et al. 2021)), a growth retardation and phenotypic variability was also observed among the rescued *ppr9* seedlings (Fig. S3b). No *ppr9* mutants could be successfully established in soil, and none of the rescued plants analyzed in this study were able to complete a full life cycle or produce viable seeds.

As the analyses of the roles of PPR9 were based on a single T-DNA insertional line (SALK_004994; *ppr9*), we generated a functional complementation line (i.e., phenotypic rescue by a functional transgene inserted at an independent genomic locus, rather than genetic complementation at the native PPR9 locus) to provide further evidence that the physiological and molecular phenotypes we see in the in vitro rescued homozygous *ppr9* mutants are indeed specifically caused by disruption of PPR9. For this purpose, the full *PPR9* coding region was cloned into the binary vector pCAMBIA-1302, under the control of CaMV-35 S promoter, and introduced into heterozygous *ppr9* plants by Agrobacterium-mediated transformation. The resulting progeny were screened to identify plants carrying the *35 S::PPR9* transgene that are also homozygous for the *ppr9* mutation (*35 S::PPR9/ppr9*). Subsequent physiological and molecular analyses involved wild type (Col-0) plants, in vitro rescued homozygous *ppr9* mutants and the complemented (Comp.) line. Importantly, as the establishment of *ppr9* required embryo rescue from white seeds collected from immature siliques, all lines analyzed here, including wild type plants, the complemented line, and when indicated also heterozygous (*PPR9/ppr9*) plants, were subjected to equivalent germination conditions (i.e., seeds collected from mature-green siliques and germinated on MS plates supplemented with sucrose and vitamins, and verified by PCR screens). Thus, the functional characterization of PPR9 roles in mitochondrial function and biogenesis was performed using different plant lines that had undergone the same in vitro germination procedure.

### PPR9 functions in the processing of *nad2* and *nad7* RNA transcripts in *A. thaliana* plants

**Fig. 4:**
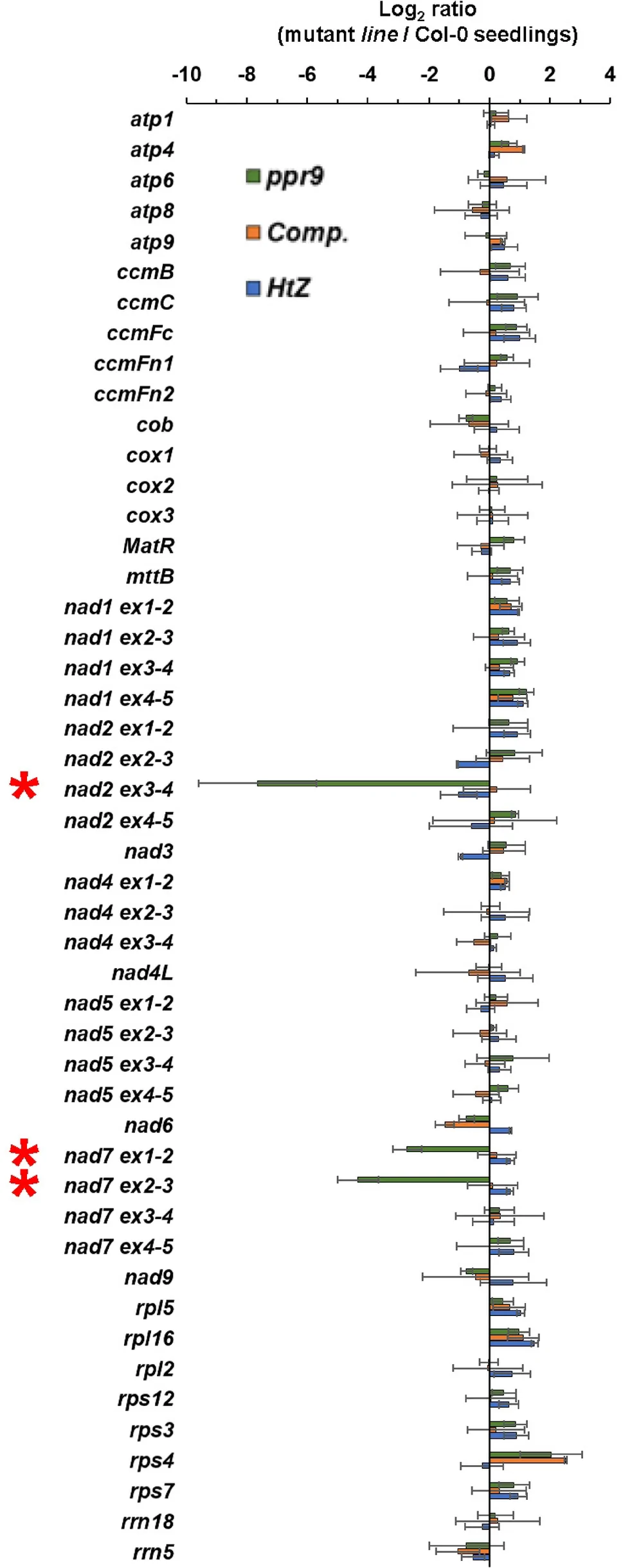
The accumulation of *nad2* and *nad7* transcripts relies on PPR9 in Arabidopsis mitochondria. The accumulation of various mitochondrial mRNA transcripts was analyzed by RT-qPCR. Total RNA was isolated from whole in vitro-grown Col-0, heterozygous (Htz), complemented (Comp.) and ppr9 seedlings, following 2 months of growth on liquid MS media. The RNA was reverse-transcribed and the relative steady-state levels of cDNAs corresponding to different organellar transcripts were estimated by qPCR with primers, which specifically amplified specific organellar transcripts (Table S4). The histograms show transcript levels in ppr9, heterozygous and complemented seedlings relative to those in Col-0. The values are means of nine RT-qPCRs corresponding to three biological replicates (error bars indicate one standard deviation), after normalization to the GAPDH (AT1G13440), ACTIN2 (AT3G18780), 18 S rRNA (AT3G41768), and the mitochondrial 26 S rRNA (rrn26, ATMG00020) genes. Red asterisks indicate transcripts whose levels were significantly reduced in the mutant relative to the wild type control

The accumulation (i.e., steady-state levels) of mitochondrial protein-coding transcripts in wild type and *ppr9* mutants was analyzed by RT-qPCR. Notably, the RNA profiles of *ppr9* plants were compared with those of germinated Col-0 seedlings derived from the same immature siliques (i.e., green seeds) and propagated in vitro under the same growth conditions. These analyses indicated a notable (~ 250-fold) reduction in processed organellar transcripts corresponding to *nad2* exons ‘3’ and ‘4’ (*nad2* ex3-4) in *ppr9* mutant plants (Fig. 4). We further noticed reduced levels in transcripts corresponding to processed *nad7* exons ‘1’ and ‘2’ (*nad7* ex1-2; about 8-fold lower) and *nad7* exons ‘2’ and ‘3’ (*nad7* ex2-3; about 20-fold lower) in *ppr9*. As the affected transcripts originated from intron-containing loci, we suspected that their altered accumulation in *ppr9* mutant plants resulted from impaired splicing of *nad2* intron 3 (*nad2* i3) and *nad7* introns 1 and 2. The steady-state levels of the organellar transcripts in the complemented (Comp.; *35 S::PPR9/ppr9*) line, including the *nad2* and *nad7* mRNAs, were comparable to Col-0 levels, with no statistically significant differences relative to the wild type plants (Fig. 4). Likewise, the accumulation of the mitochondrial transcript in heterozygous (Htz) plants were similar to those of the Col-0 plants (Fig. 4). Taken together, the RT-qPCR data of mitochondrial protein-coding transcripts indicate a key role for PPR9 in the maturation of *nad2* and *nad7* transcripts.

#### PPR9 is required for the efficient splicing of *nad2* intron 3 and *nad7* introns 1 and 2

Based on the organellar mRNA profiles and reduced steady-state levels of *nad2* ex3-4 and *nad7* ex1-2 and ex2-3 transcripts in *ppr9* seedlings (Fig. 4), we considered defects in the splicing of *nad2* i3 and *nad7* introns 1 and 2, respectively. To test this assumption, we quantified the splicing efficiencies of the 23 mitochondrial introns in Arabidopsis mitochondria. Splicing efficiency was evaluated by RT-qPCR as the ratio of specific mRNAs (excised) to their corresponding pre-RNAs (unspliced) in mutant lines relative to the wild type seedlings. A specific splicing defect was inferred only when accumulation of an intron-containing precursor in the mutant was accompanied by a corresponding reduction in the mature mRNA, thereby distinguishing impaired splicing from changes in transcript abundance that may arise independently of RNA processing.

**Fig. 5:**
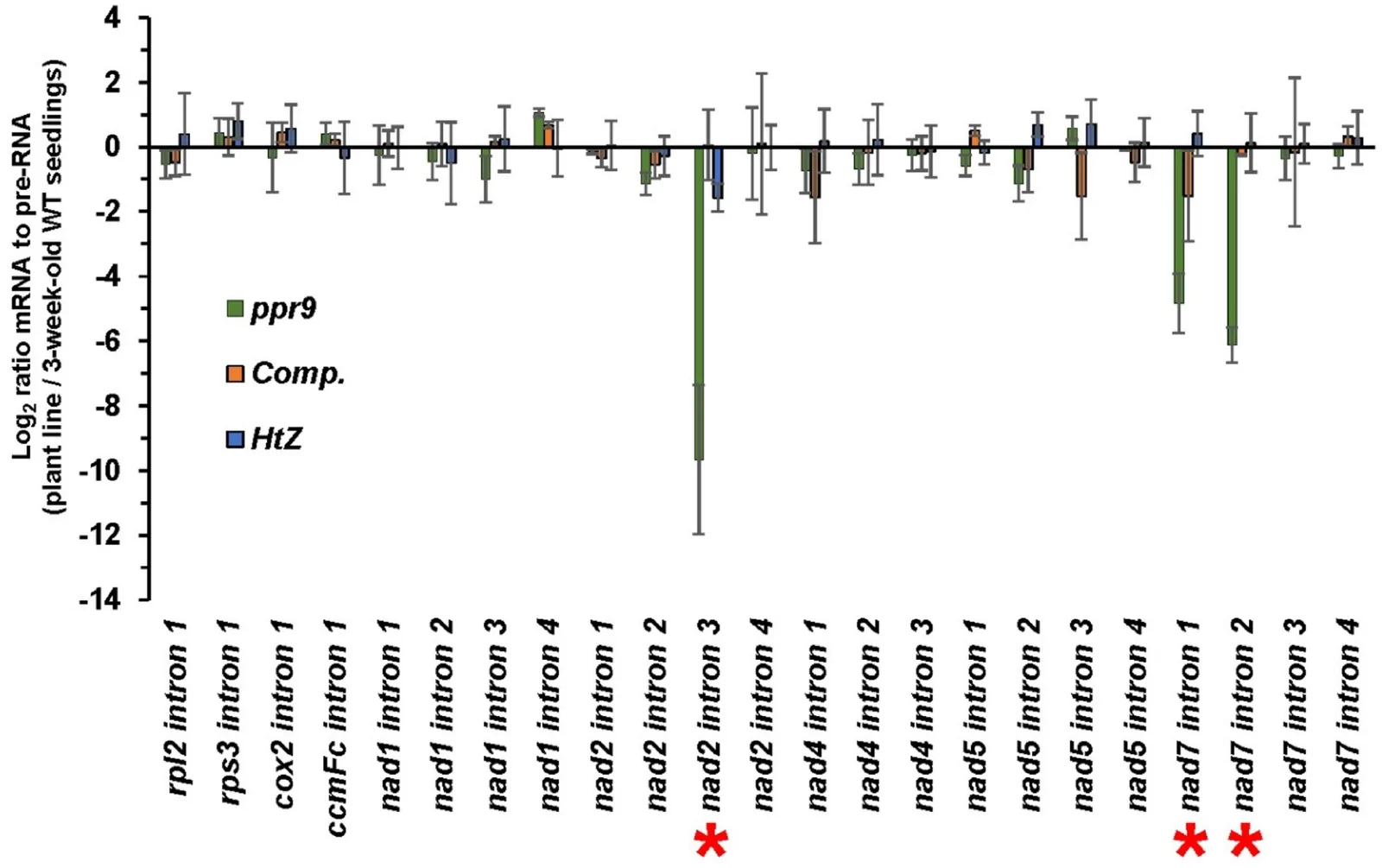
PPR9 functions in splicing of *nad2* intron 3 and *nad7* introns 1 and 2 in Arabidopsis mitochondria. Total RNA was extracted from whole in vitro-grown Col-0, heterozygous (Htz), complemented (Comp.) and ppr9 seedlings, following 2 months of growth on liquid MS media. The RNA was reverse-transcribed and the relative steady-state levels of cDNAs corresponding to different organellar transcripts were estimated by qPCR with primers, which specifically amplified specific organellar transcripts (Table S4). The splicing efficiencies of the 23 mitochondrial group II introns were assessed based on the relative mRNA to pre-RNA ratios (presented as the log2) in mutant lines compared with those of wild type plants. The histograms show transcript levels in ppr9, heterozygous and complemented seedlings relative to those in Col-0. For quantification, transcript levels were further normalized to various reference genes, including the GAPDH (AT1G13440), ACTIN2 (AT3G18780), 18 S rRNA (AT3G41768), and the mitochondrial 26 S rRNA (rrn26, ATMG00020) genes. Data represent mean values from three biological replicates, each analyzed in triplicate (nine reactions in total). Error bars represent one standard deviation

An impairment in mitochondrial splicing was evident for *nad2* i3, with about 1000-fold reduction in splicing efficiency in *ppr9* relative to Col-0 plants (Fig. 5). The splicing efficiencies of *nad7* introns 1 and 2 were also affected, although to a lesser extent, showing approximately 30- and 60-fold reductions, respectively (Fig. 5). While some Arabidopsis mutants defective in mitochondrial intron splicing may show increased accumulation of other organellar transcripts, other than the splicing defects seen in *nad2* intron 3 and *nad7* introns 1 and 2, the splicing efficiencies of other mitochondrial introns in *ppr9* were largely comparable to those observed in Col-0 plants, as determined by the RT-qPCR analyses (Fig. 5). The processing of the organellar transcripts in heterozygous (Htz) and the complemented (Comp.) lines were comparable to those of Col-0 plants (Fig. 5). These data indicate a role for PPR9 in the maturation of three group II-type introns in *A. thaliana* mitochondria, including *nad2* i3 and *nad7* introns 1 and 2.

The RNA profiles were further analyzed by RNase-protection assays, generally as described previously (Cohen et al. 2014; Keren et al. 2009, 2012; Ostersetzer et al. 2005). Complementary RNA probes, generated by in vitro transcription, were designed to hybridize to exon-exon junctions (to detect mature transcripts; ‘m’) or exon-intron/intron-exon junctions (to detect unspliced pre-RNAs; ‘p’). The RNA probes were incubated with total RNA isolated from wild type (Col-0), *ppr9* and complemented (Comp.) plants, followed by treatment with RNase T1. These assays also indicated to altered processing (i.e., accumulation of pre-RNAs followed by a reduction in the corresponding mRNA) of *nad2* i3 and *nad7* introns 1 and 2 in *ppr9* (Fig. S4). The splicing efficiencies of other intron-containing transcripts, including *cox2* and *rpl2*, as well as the accumulation of an “intronless” transcript (i.e., *nad6* mRNA), appeared to be unaffected (Fig. S4). However, we observed an accumulation of pre-RNA corresponding to *nad2* i2 in the *ppr9* mutant, a *trans*-spliced transcript whose precursor was also found to accumulate by RT-qPCR (Fig. S4). Since the processing of transcripts corresponding to matured *nad2* exon 2–3 RNAs showed only minor changes in *ppr9* (Figs. 4, 5, and S4), we speculate that an accumulation of pre-RNAs corresponding to the 5′ part of *nad2* i2 (contains ex1, i1, ex2 and the 5′-part of i2; Fig. S4) in *ppr9* likely results from the sequential nature of *nad2 trans*-splicing, where impaired processing of *nad2* i3 (found on the second transcript unit of *nad2*; i.e., harboring the 3′-end of i2, ex3, i3, ex4, i4 and ex5) affects the processing of its related product, the precursor the first transcript unit of *nad2* (Fig. S4). Accordingly, in genes containing multiple introns, particularly those harboring *trans*-spliced introns (i.e., *nad1*, *nad2*, and *nad5*), the RNA processing steps seem to be interdependent. For example, the splicing or availability of one exon-intron segment can influence the substrate for the downstream step, as in the cases of e.g., OTP43 (Falcon de Longevialle et al. 2007) or MSP1 (Best et al. 2023), where defects in *nad1* i1 processing also affect ‘downstream’ RNA processing steps.

### PPR9 deficiency disrupts the assembly and function of mitochondrial complex I (CI)

The OXPHOS system consists of five major protein complexes, CI (~ 1000 kDa), CII (~ 160 kDa), a dimeric complex III (CIII_2_, ~ 500 kDa), CIV (~ 220 kDa), and the ATP synthase enzyme (CV, ~ 660 kDa). The sequential incorporation of NAD2 and NAD7 subunits is an essential for the assembly of a functional holo-CI in plants (Braun 2020; Ligas et al. 2019; Meyer et al. 2019; Ostersetzer-Biran 2016). The reductions we see in the splicing efficiencies of several *nad2* and *nad7* pre-RNA transcripts (Figs. 4, 5) suggest that their corresponding subunits may be present at lower levels in *ppr9* mutants. The relative accumulation of mitochondrial complexes in enriched organellar membranous fractions, obtained from Col-0, in vitro rescued homozygous *ppr9* mutants (Hmz) and the complemented (Comp.) line were analyzed by blue-native (BN-) PAGE, followed by immunoblotting with antibodies against various organellar protein subunits.

**Fig. 6:**
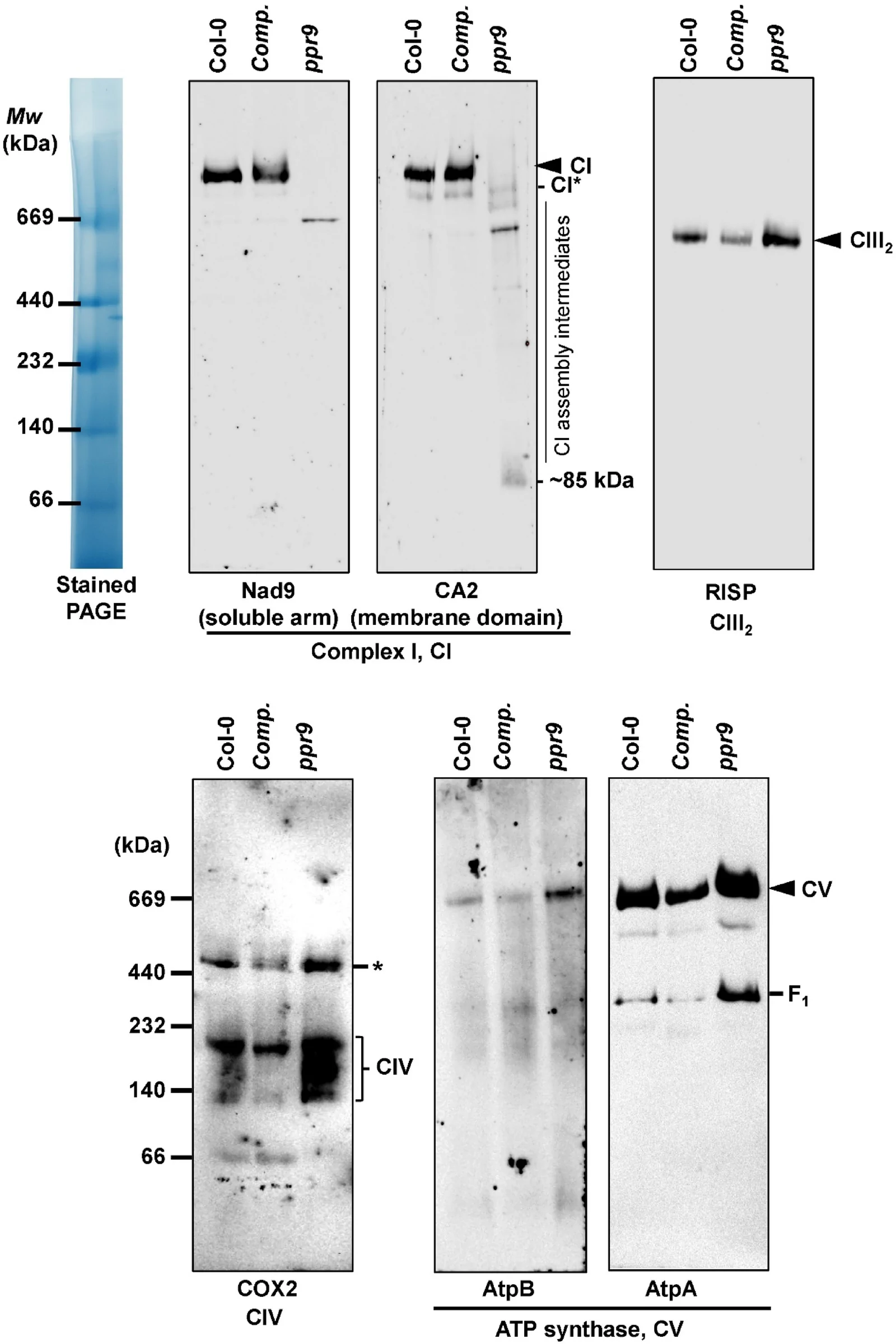
Defects in mitochondrial respiratory CI biogenesis in *ppr9* mutants. Mitochondrial respiratory complexes were analyzed in mitochondria-enriched membrane fraction prepared from 2-month-old plants grown on liquid MS-media. The abundance and assembly state of mitochondrial respiratory complexes were assessed by blue-native polyacrylamide gel electrophoresis (BN-PAGE) of mitochondria-enriched membrane preparations from Col-0 plants and ppr9 mutant lines. Following native electrophoresis, proteins were transferred to PVDF membranes and analyzed by immunoblotting using antibodies against various organellar protein subunits (Table S5), following chemiluminescence (ECL) assays and then recorded on an imaging system (ImageQuant LAS 4000, GE Healthcare, Haifa, Israel). Signal detection was performed by chemiluminescence. Arrows indicate the positions of the major native respiratory complexes, including NADH: ubiquinone oxidoreductase (CI; ~1000 kDa), cytochrome bc1 complex dimer (also called ubiquinol: cytochrome c oxidoreductase, or CIII_2_; ~500 kDa), cytochrome c oxidase (CIV; ~220 kDa), and the ATP synthase enzymes (CV; ~660 kDa). The asterisk in the CA2 panel marks the 800 ~ 850 kDa CI stable assembly intermediate (CI*), while additional lower-molecular-mass particles in the CA2 blot correspond to various CI-assembly intermediates of the membrane domain (Ligas et al. 2019; Ostersetzer-Biran 2016). The asterisk in the COX2 blot marks a ~450–500-kDa particle, previously observed in another CI-deficient mutant plant (Shevtsov-Tal et al. 2021)

The BN-PAGE analysis revealed to altered CI biogenesis in *ppr9* plants. This was apparent by immunoblotting with antibodies against the CA2 that is embedded within the membranous domain of CI and the Nad9 protein of the soluble arm of CI, in which the levels of holo-CI (i.e., ~ 1000 kDa) were notably reduced (bellow detectable levels) in *ppr9* (Figs. 6 and S5). The stable assembly intermediate of CI (~ 800–850 kDa; CI*), which contains many subunits of the soluble and membrane domains (Ligas et al. 2019), is indicated. We further noticed the accumulation of various protein bands of lower molecular masses in the CA2 in *ppr9* plants (Figs. 6 and S5). Similar CI-assembly intermediates of the membranous domain have been also reported in other Arabidopsis mutants defective in CI biogenesis (Braun et al. 2014; Kuhn et al. 2015; Ligas et al. 2019; Meyer et al. 2019; Ostersetzer-Biran 2016). A ~ 700 kDa CI-assembly intermediate was detected in the Nad9 immunoblots (Figs. 6 and S5), that is possibly related to impaired assembly of the soluble arm of CI due to altered Nad7 expression (Figs. 4, 5). The relative abundance of native CI in the heterozygous line was comparable to that of the wild type plants (Fig. S5).

While CI was notably reduced in *ppr9*, the BN-PAGE rather revealed increased steady-state levels of dimeric complex III (CIII_2_, detected with antibodies against the Rieske Fe-S protein, RISP); complex IV (CIV, detected with anti-COX2 antibodies as a diffuse, smeared signal) and ATP synthase (CV, detected with anti- AtpA and AtpB antibodies) (Fig. 6). The relative steady-state abundances of native organellar complexes were estimated by densitometric analysis of the immunoblot signals using Fiji (Schindelin et al. 2012) and are presented in Table S2a as approximate ratios relative to wild type signals.

The immunoblots also revealed to additional CIV- and CV-related bands (Fig. 6). The COX2 blot showed a 450 ~ 500 kDa particle (labeled with asterisk), previously seen in other CI-deficient plants, that may represent a CIV dimer or aberrant CIV assembly particles (Shevtsov-Tal et al. 2021). The AtpA and AtpB blots indicated the presence of the F_1_ ATP-synthase module (Senkler et al. 2017). The signals of the COX2-associated particle and the F_1_ module also appeared higher in *ppr9*. The abundance of ribulose-1,5-bisphosphate carboxylase/oxygenase (Rubisco), a key photosynthetic enzyme, was similar to that of Col-0 plants (Fig. S5 and Table S2a).

As shown by BN-PAGE, native CI levels in the complemented (Comp.) line of *ppr9* mutants were clearly increased relative to those in the homozygous mutants, where holo-CI was below the detection limit (Figs. 6, S5 and Table S2a). The complemented line retained lower levels of holo-CI and several other respiratory complexes, including CIII_2_, CIV, and CV, relative to Col-0, whereas the native Rubisco complex was slightly more abundant (Fig. S5 and Table S2a). As equal fresh weights may differ in tissue composition and mitochondrial content, the reduced complex signals in the complemented line may not solely reflect incomplete complementation. Nevertheless, the reappearance of holo-CI demonstrates a restoration of holo-CI biogenesis. The observed differences from Col-0 may also result from ectopic PPR9 expression driven by the CaMV-35 S promoter rather than its own native promoter.

**Fig. 7:**
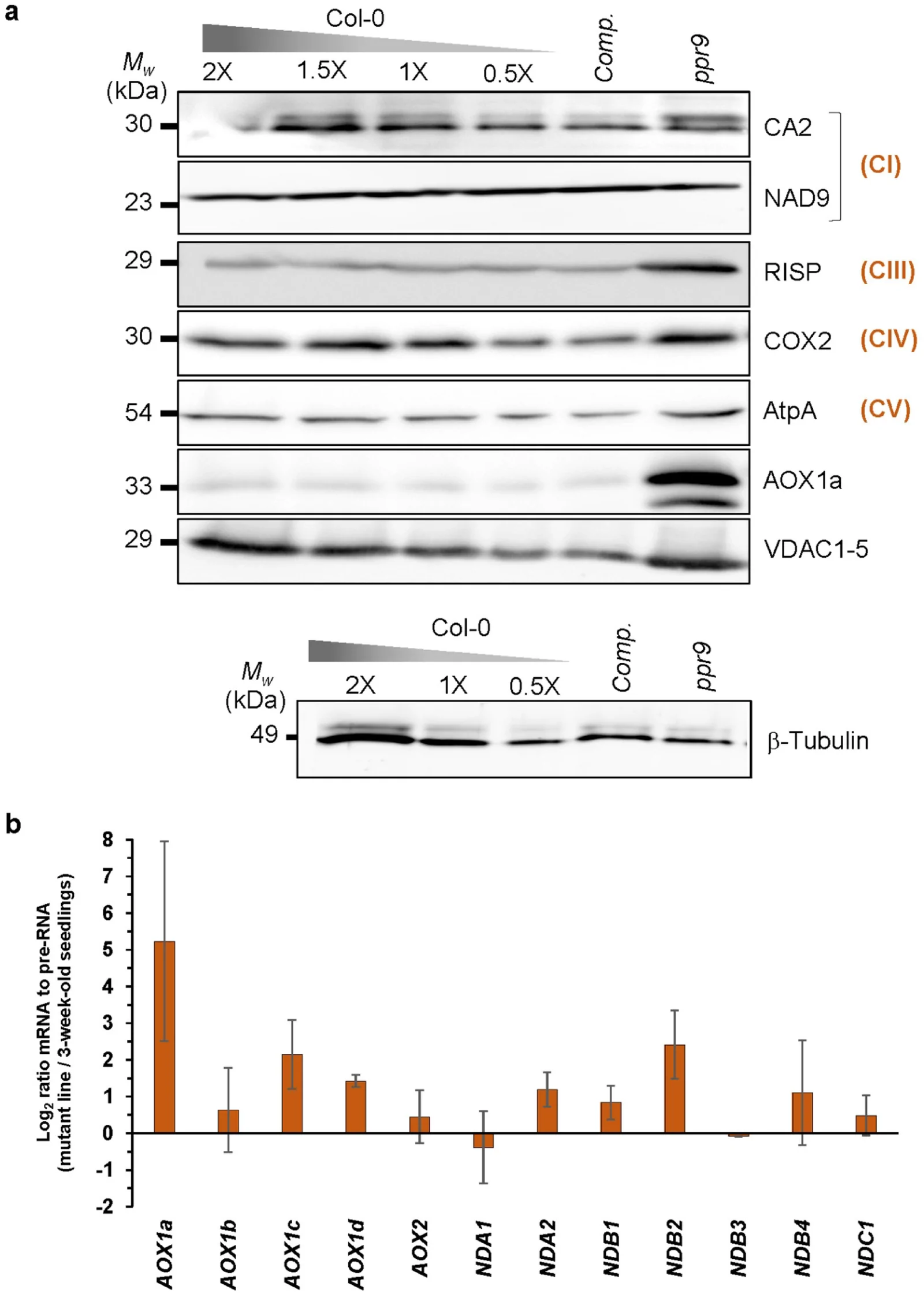
Accumulation of various organellar proteins in wild type and *ppr9* mutant plants, and induction of the alternative electron transport pathway. **a** mitochondria-enriched membrane fractions were prepared from 2 month-old plants grown in liquid MS-medium. Equivalent amounts of protein were separated by SDS-PAGE and analyzed by immunoblotting with specific antibodies raised against representative organellar proteins (Table S5). Signals were detected using chemiluminescence (ECL) and recorded on an imaging system. **b** Analysis of the steady-state levels of alternative oxidases (AOXs) and rotenone-insensitive NAD(P)H dehydrogenase (NDs) transcripts by RT-qPCR. The histogram shows transcript levels in ppr9 relative to those in Col-0 seedlings grown for 2 months liquid MS culture conditions. For quantification, transcript levels were further normalized to various reference genes, including the GAPDH (AT1G13440), ACTIN2 (AT3G18780), 18 S rRNA (AT3G41768), and the mitochondrial 26 S rRNA (rrn26, ATMG00020) genes. Data represent mean values from five biological replicates, each analyzed in triplicate (15 reactions in total). Error bars represent one standard deviation

The steady-state levels of various mitochondrial proteins were examined by denaturing SDS-PAGE, followed by immunoblotting. Relative protein abundances were estimated by Fiji densitometry (Schindelin et al. 2012), using a series of wild type loading amounts as references and normalizing the protein signals to β-tubulin. Based on these assays, CA2 abundance was only slightly reduced in *ppr9* relative to Col-0 (Fig. 7a and Table S2b). An additional band of higher size was detected with the anti-CA2 antibody, most likely reflecting cross-reactivity with the related CA1 or CA3 proteins (Perales et al. 2005; Sunderhaus et al. 2006). NAD9 was also detected at similar levels to those of the wild type plants, although its abundance could not be reliably quantified. RISP abundance was markedly increased in *ppr9*, about 3-fold higher that in Col-0 seedlings (Table S2b). COX2 and AtpA showed moderate increases, whereas VDAC1-5 signal was about 2-fold higher in *ppr9*.

In plant mitochondria, various alternative oxidases (AOXs) and type II NAD(P)H dehydrogenases (NDs) provide alternative routes for electron flow that bypass components of the classical cytochrome respiratory pathway. The strongest effect was noted for AOX1a, which accumulated in *ppr9* to about 20 folds of the wild type levels (Fig. 7a and Table S2b). In the complemented line, CA2 abundance was slightly reduced (~ 0.8-fold) and COX2 was similar to Col-0, whereas AtpA, AOX1a, RISP, and VDAC accumulated to levels approximately 1.4- to 1.7-fold higher than in Col-0.

In addition to the immunoblot analyses, we also examined the expression of genes encoding various AOX and ND proteins by RT-qPCR. This analysis further revealed increased transcript levels of *AOX1a*, which exhibited the strongest induction, as well as higher levels in transcripts corresponding to *AOX1c*, *AOX1d*, *NDA2*, and *NDB2* in *ppr9* mutants (Fig. 7b). Milder (statistically non-significant) changes in transcript levels were also seen for *AOX1b*, *AOX2*, *NDA1*, *NDB4* and *NDC1* (Fig. 7b).

**Fig. 8:**
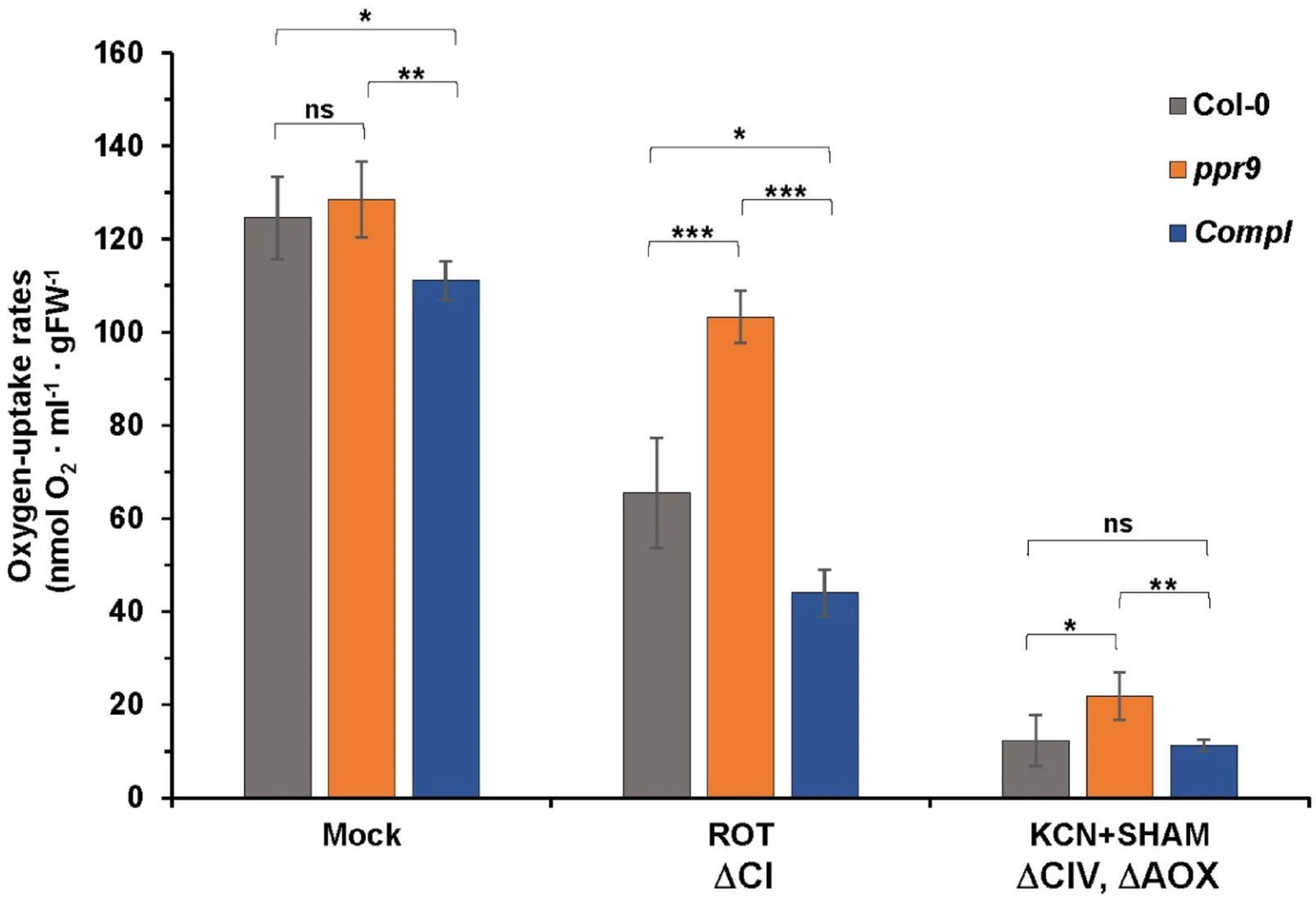
Respiratory activity in wild type, *ppr9* and complemented plants. Oxygen (O_2_) consumption rates were measured in ~ 2-month-old liquid MS-grown wild type (Col-0), *ppr9* and complemented (Comp.) plants under control conditions, and following inhibition of mitochondrial CI with rotenone (ROT; 50 µM), and the combined inhibition of the cytochrome and alternative respiratory pathways with KCN (1.0 mM) and SHAM (1.0 mM), respectively. Values represent means ± SD from five biological replicates. Statistical significance between genotypes within each treatment was determined using two-sided unpaired Student’s t-tests with Holm–Šídák correction for multiple comparisons. ns, not significant; *P* ≥ 0.05; * *P* < 0.05; ** *P* < 0.01; *** *P* < 0.001

The RNA and protein profiles suggested to altered mitochondria respiratory functions. The O_2_-uptake rates of wild type (Col-0), *ppr9* and complemented (Comp.) plants were analyzed by a Clark-type electrode. The respiration rates of *ppr9* seedlings (129 ± 8 nmol O_2_ min^−1^ g FW^−1^) were similar, of even slightly increased (not statistically significant) to that of Col-0 plants (125 ± 9 nmol O_2_ min^−1^ g FW^−1^) (Fig. 8). The respiratory activity was further measured in the presence of various electron-transport inhibitors, including rotenone (ROT; 50 µM), a specific inhibitor of CI, and under the combined inhibition of potassium cyanide (1 mM KCN; CIV inhibitor) and salicylhydroxamic acid (1 mM SHAM; AOX inhibitor). As indicated in Fig. 8, rotenone had a notable effect (i.e., 47% inhibition) on the respiration of wild type plants, in which the O_2_-uptake rates (66 ± 12 nmol O_2_ min^−1^ g FW^−1^) were notably lower than the untreated plants (Mock). Yet, the addition of ROT had a much milder effect on *ppr9* respiration, reducing the O_2_-uptake rates by ~ 20% (i.e., 103 ± 6 nmol O_2_ min^−1^ g FW^−1^ ). The addition of KCN and SHAM strongly affected the respiration activity in the wild type and mutant plants to nearly basal levels, i.e., 12 ± 6 nmol O_2_ min^−1^ g FW^−1^ (~ 90% inhibition) in Col-0, and 22 ± 5 nmol O_2_ min^−1^ g FW^−1^ (~ 85% inhibition) in *ppr9* (Fig. 8). Similar to wild type plants, respiration in the complemented line was inhibited by about 60% by ROT and more profoundly (~ 90%) by the combined effects of KCN and SHAM (Fig. 8).

## Discussion

### *PPR9* encodes a mitochondrial P-type PPR protein essential for early embryogenesis

Mitochondria are central hubs of eukaryotic energy metabolism and hence are indispensable for plant physiology. Expression of mitochondrial genes in land plants requires numerous post-transcriptional processing steps, including the splicing of group II-type introns that are embedded within the coding regions of many essential mitochondrial genes (Colas des Francs et al. 2014; Hammani and Giege 2014; Small et al. 2023; Zmudjak and Ostersetzer-Biran 2018). These complex mt-RNA processing steps rely on a large and diverse set of RNA-binding proteins that may also serve as major regulatory cofactors for mitochondria biogenesis, respiratory activity, and consequently, plant growth and development. Here, we describe the molecular and biochemical analysis of PPR9, an essential P-type PPR protein factor in *A. thaliana* plants, encoded by the *AT1G03560* gene-locus.

Based on available databases (e.g., TAIR, UniProt, SUBA5) At.PPR9 encodes a lowly expressed mitochondria-targeted protein, harboring 13 PPR motifs (Figs. 1, S1). Publicly available proteomic data (Fuchs et al. 2020) and our GFP localization analysis (Fig. 2) support the in-silico predictions and demonstrated that PPR9 reside in mitochondria in vivo. An embryo-rescue in vitro method (Figs. 3, S3) (Shevtsov-Tal et al. 2021) allowed the establishment of homozygous *ppr9* (SALK_004994) mutant plants under in vitro conditions, which their RNA and protein profiles indicated that PPR9 is pivotal for mt-RNA processing, and hence for mitochondria OXPHOS biogenesis (Figs. 4, 5 and S4). Altered expression of Nad2 and Nad7 in *ppr9* mutants affects respiratory CI biogenesis (Figs. 6, 7), which is strongly associated with defects in embryogenesis and seed development in the *ppr9* mutant Arabidopsis plants (Figs. 3 and S3).

Regulation of PPR9 expression or protein abundance (i.e., through proteolytic turnover), may provide with additional layer of nuclear control of organellar gene expression. In Drosophila mitochondria, ClpXP-mediated degradation of a PPR protein (Dm.LRPPRC1) was suggested to modulate mitochondrial transcript abundance, RNA processing or translation (Matsushima et al. 2017), whereas mammalian LRPPRC was suggested to be protected from LONP1-mediated degradation through its association with SLIRP (Lagouge et al. 2015). In plants, Clp-dependent turnover has been indicated for the plastidial GUN1 protein (Wu et al. 2018). However, whether proteolytic regulation similarly controls the abundance and activity of mitochondrial PPR splicing factors in plants remains unknown.

### PPR9 functions in the splicing of several *nad2* and *nad7* group II-type introns

The mitochondrial introns in Arabidopsis mitochondria belong to the group II intron family (Bonen 2008; Brown et al. 2014; Lambowitz and Belfort 2015; Schmitz-Linneweber et al. 2015; Zimmerly and Semper 2015). Unlike canonical group II introns, whose splicing is facilitated by intron-encoded proteins (IEPs, or maturases) (Lambowitz and Belfort 2015; Lambowitz and Zimmerly 2011; Mizrahi et al. 2022; Zhao and Pyle 2017), the splicing of mitochondrial introns in plants relies on a large and diverse set of nuclear-encoded protein cofactors. Some, such as PPR proteins, act specifically on a single intron target, whereas others, including Arabidopsis maturases, RNA helicases, and CRM- or PORR-related factors, function in the splicing of multiple introns (Bonen 2008; Brown et al. 2014; Schmitz-Linneweber et al. 2015; Small et al. 2023; Zmudjak and Ostersetzer-Biran 2018). PPR proteins constitute a particularly large family in angiosperms, comprising ~ 450 members in Arabidopsis (Barkan and Small 2014; O’Toole et al. 2008; Schmitz-Linneweber and Small 2008; Small and Peeters 2000). These are postulated to bind single-stranded RNA in a modular and sequence-specific manner, and were shown to play key roles in organellar RNA metabolism, such as in RNA stabilization, editing, cleavage, and group II intron splicing (Small et al. 2020, 2023; Takenaka et al. 2019; Zmudjak and Ostersetzer-Biran 2018), and potentially also in mitoribosome biogenesis or translation (Kwasniak-Owczarek et al. 2019; Rugen et al. 2019; Waltz et al. 2019). Likewise, genetic characterization of *ppr9* mutants revealed a notable reduction in the steady-state levels of processed mitochondrial transcripts corresponding to *nad2* exons 3–4 and *nad7* exons 1–2 and exons 2–3 (Figs. 4 and S4). RT-qPCR and RNase-protection assays further indicated that the removal of intron 3 of *nad2* and introns 1 and 2 of *nad7* was strongly affected in the mutant (Figs. 5 and S4).

In some cases, in-silico PPR RNA-binding predictions (Barkan and Small 2014; Cheng et al. 2016; Gully et al. 2015; Shen et al. 2015; Yan et al. 2019) identify specific RNA-binding sites that correspond to experimentally validated biological targets. Yet, the current PPR code did not revealed a clear and specific binding site for PPR9. Instead, more than 200 putative binding sites were predicted for PPR9 across exonic, intronic and UTR sequences (excluding intergenic regions) (Table S1). These included *nad2* intron 3, and *nad7* introns 1 and 2 (Fig. 1B and Table S1), the three validated targets of PPR9, which appear to share common binding chrematistics. The molecular analysis of PPR9 suggests a refined binding code for PPR motifs that may improve the accuracy of identifying the in vivo targets of other PPR proteins. For example, the amino acids Met and Pro at positions 5 and 35, respectively, are predicted to bind A, U, or G; Arg and Asp may confer specificity for A; Asp and Pro are associated with binding to A or G ribonucleotides; and Thr and Met are linked to A, G, or U recognition. We also observed a single mismatch to the predicted consensus for the Thr-Thr combination at positions 5 and 35 of the 10th PPR motif, where a C residue occurs instead of A or G (Fig. 1B).

### PPR9 functions are critical for holo-CI biogenesis and embryo development

The mitochondrial electron transport chain (ETC) comprises of four main respiratory complexes (CI-CIV) that generate a proton gradient across the inner membrane, driving ATP biosynthesis by the ATP synthase enzyme (also denoted as CV) (Braun 2020; Millar et al. 2011; Møller et al. 2021). In addition to the canonical respiratory complexes, plants (and several other organisms) possess AOXs and NDs that can bypass the canonical ETC system (Braun 2020; Møller et al. 2021). CI, the largest respiratory complex (~ 1000 kDa), is composed of about 50 subunits, derived from expression of several mitochondrial gene-loci (*nad1*, *nad2*, *nad3*, *nad4*, *nad4L*, *nad5*, *nad6*, *nad7* and *nad9*) and numerous nuclear-encoded subunits that are imported into the organelle (Klusch et al. 2023; Møller et al. 2021). These are assembled together in the organelles into two main subdomains (i.e., membrane-arm and the matrix-associated domain) that together form the functional holoenzyme, which is further assembled into respiratory chain supercomplexes (respirasomes), e.g., (Klusch et al. 2023).

NAD2 and NAD7 subunits play pivotal roles in assembling the membrane domain and the matrix arm, respectively (Ligas et al. 2019). Accordingly, the *ppr9* mutants that are affected in the maturation of *nad2* and *nad7* transcripts exhibited a notable reduction in holo-CI levels, as revealed by BN-PAGE (Figs. 6 and S5). Immunoblotting with anti-CA2 antibodies further revealed several distinct complex I-related protein bands, including an ~ 85 kDa particle (Fig. 6, CA2 panel), previously observed in *abo5* (Ligas et al. 2019; Liu et al. 2010) and *misf2* (Nguyen et al. 2022) mutants, both of which are defective in *nad2* pre-RNAs processing. These particles likely represent low-molecular-mass CI assembly intermediates lacking the NAD2 subunit, which is incorporated early into the membranous arm of CI, and hence various other CI subunits that are incorporated at later CI-assembly stages (Ligas et al. 2019).

Another notable feature of the *ppr9* mutant was the notable accumulation of AOX1a (Fig. 7a), indicative of activation of the alternative respiratory pathway. CI biogenesis defects in land plants are typically accompanied by the induction of alternative electron-transport pathways, including the rotenone-insensitive NDs and the cyanide-resistant AOXs (Braun 2020; Møller et al. 2021). This was also evident in *ppr9* mutants by RT-qPCR data, which indicated the upregulation in the expression of various *AOX* and *ND* genes (i.e., *AOX1a*, *AOX1c*, *AOX1d*, *NDA2*, *NDB1*, *NDB2* and *NDB4*; Fig. 7b). Such responses that seem hallmark of OXPHOS deficient mutants in plants can be attributed as part of the mitochondrial retrograde signaling that may assist in maintaining electron flux and minimizes excessive ROS effects (Braun 2020; Kuhn et al. 2015; Møller et al. 2021). Likewise, the protein profiles (Figs. 6, 7 and S5) and respiratory analysis (Fig. 8) supported a reduced CI that is followed by the increase and remodeling of the electron transport system and ATP-synthase complex.

Taken together, these data indicate that CI biogenesis and activity are impaired in *ppr9*. The reduced sensitivity of *ppr9* respiration to rotenone, together with the induction of alternative respiratory components, seem to align well with increased dependency on alternative electron-transport pathways (Braun 2020; Kuhn et al. 2015; Møller et al. 2021). Similar compensatory remodeling has been reported in other Arabidopsis mutants impaired in CI biogenesis (Frink et al. 2024; Kobaivanov et al. 2026; Nguyen et al. 2022), although it seems not universal among CI-deficient mutants (Kühn et al. 2011). The response may therefore depend on the nature of the genetic defect and its physiological consequences and may help maintain electron transport and cellular energy and redox homeostasis when CI activity is compromised (Kuhn et al. 2015).

### PPR9-mediated maturation of mitochondrial *nad* transcripts and CI biogenesis are critical for embryo development

Mutant plants defective in mitochondrial function, as well as those with impairments in cellular metabolism, such as fatty acid β-oxidation (Pinfield-Wells et al. 2005), display abnormal embryo development phenotypes, often with a ‘Wrinkled’ seed morphology (Pinfield-Wells et al. 2005). Impaired CI biogenesis is likewise tightly associated with defective embryogenesis and subsequent growth and developmental abnormalities (Best et al. 2020; Ostersetzer-Biran 2016), where the degree of CI-deficiency has previously been correlated with phenotypic severity (Colas des Francs et al. 2014; Kuhn et al. 2015; Ostersetzer-Biran 2016). These abnormal physiological effects likely result from impaired cellular functions and reduced nutrient reserves in the seed (Pinfield-Wells et al. 2005). Indeed, the *ppr9* mutants exhibited early embryonic arrest, with *wrinkled* seed-morphology, failing to germinate under ‘standard’ (in vivo) growth conditions (Figs. 3a and S3).

In some cases, germination and seedlings establishment can be rescued under in vitro conditions, using immature seeds typically germinated on enriched growth media. The precise molecular mechanism underlying embryo- and seedlings growth and development rescue is still not fully understood. It is possible that the additional sugars, minerals and vitamins can support essential metabolic functions that compensate for metabolic constraints in mitochondrially affected mutant embryos (Dahan et al. 2014; Shevtsov-Tal et al. 2021). Also, prematurely harvested seeds with a softer seed coat may allow the emergence of the seedlings radicle and hypocotyl, by increasing permeability and reducing mechanical resistance (Huss and Gierlinger 2021). The few germinated *ppr9* mutant plants further displayed arrested growth and other phenotypes (Figs. 3c and S3), which are characteristic of CI-deficient plants (Colas des Francs et al. 2014; Kuhn et al. 2015; Ostersetzer-Biran 2016). While exogenous sugars and vitamins supported limited vegetative development, the mutants likely remained metabolically compromised, as they were unable to complete their life cycle or produce viable seeds. These findings highlight the pivotal roles of PPR9-mediated mt-RNA processing in OXPHOS biogenesis and respiratory function and, consequently, in normal plant development and physiology.

## Materials and methods

### Plant material and growth conditions

The experiments involved *Arabidopsis thaliana* (Col-0) plants. Seeds of the wild type and the *ppr9* mutants (SALK_004994) were obtained from the Arabidopsis Biological Resource Center (ABRC, Ohio State University, USA). For germination on half-strength (1/2) Murashige and Skoog (MS)-agar medium (M0221, Duchefa Biochemie, Haarlem, Netherland) wild type and mutant seeds were sterilized with Cl_2_ gas and sown on MS-agar plates and rescued using the method detailed below. After a 5-day stratification period at 4 °C, the plates were transferred to a growth chamber (Percival Scientific, Perry, IA, USA) set to 22 °C with long-day conditions (16 h light/8 h dark), RH 50%, with a light intensity of 100 ~ 150 µE m^−2^ s^−1^. The identity and integrity of each line were confirmed by PCR genotyping using gene-specific and T-DNA-specific primers (Table S3). Seeds from confirmed lines were sterilized, germinated on MS-agar medium containing 1% sucrose, and analyzed for their associated genetic and biochemical phenotypes. Unless indicated otherwise, molecular and biochemical analyses were performed using ~ 2 month-old in vitro-grown (whole) seedlings, obtained from MS-agar plates and transferred to liquid MS medium supplemented with sucrose and vitamins (see ‘In vitro rescue of homozygous ppr9 mutants’), with moderate shaking (50–100 rpm) (Shevtsov-Tal et al. 2021).

### In vitro rescue of homozygous *ppr9* mutants

Embryo rescue of the *ppr9* mutant line was performed essentially as described previously (Best et al. 2023; Shevtsov-Tal et al. 2021). In brief, mature green siliques from heterozygous *ppr9* plants were surface-sterilized and then rinsed thoroughly with sterile DDW. Green (wild type or heterozygous; *PPR9/ppr9* ) and white (homozygous; *ppr9/ppr9*) seeds isolated from the sterilized siliques were sown on MS-agar plates supplemented with 1% (w/v) sucrose and a vitamin mix (i.e., 100 µg myoinositol, 1.0 µg thiamine, 1.0 µg pyridoxine, 1.0 µg nicotinic acid per liter of media). To obtain larger quantities of plant material, required for BN-PAGE analyses, seedlings at early developmental stages (L4 to L6 rosette leaves) (Boyes et al. 2001) were transferred to a liquid MS medium containing 1% sucrose and the same vitamin mix and grown under the conditions described previously, with gentle agitation (50–100 RPM), as we performed previously (Best et al. 2023; Shevtsov-Tal et al. 2021). For complementation of the *ppr9* mutation, the full coding sequence of *PPR9* was cloned into pCAMBIA-1302 (CAMBIA, Canberra, Australia; accession AF234298.1) under the control of the CaMV-35 S promoter and the NOS terminator. The resulting (untagged) construct, containing the full-length PPR9 coding sequence under the control of the 35 S promoter, was introduced into heterozygous (Htz., *PPR9/ppr9*) plants by *Agrobacterium*-mediated transformation to generate complemented (Comp.) *ppr9* lines, which are homozygous for the *ppr9* mutation and carrying the *35 S::PPR9* transgene (Fig. S3a).

### Imaging-based analysis of Arabidopsis plant tissues

For morphological analyses, plant tissues were examined using a stereoscopic (dissecting) microscope or a light microscope at the Bio-Imaging Unit, Institute of Life Sciences, The Hebrew University of Jerusalem.

### Protoplast preparation and GFP localization assays

GFP fusion proteins were expressed in protoplasts prepared from *Physcomitrium patens* (Thévenin et al. 2012, 2016), and transformed generally as described previously (Charlot et al. 2022). In brief, protoplasts were obtained from harvested tissue of a 1-month-old liquid culture (protonemata enriched) and treated with 4% (w/v) Driselase in 0.5 M mannitol. The resulting suspension was filtered through a 45 μm sieve. A DNA product encoding the first 82 amino acids of the intronless *PPR9* gene, fused in-frame to eGFP, was cloned into the binary pCAMBIA-1302 vector (CAMBIA, Canberra, Australia; https://www.cambia.org; GenBank AF234298.1) under the control of CaMV 35 S promoter and a NOS terminator. The pCAMBIA-1302 containing 82 amino acids of the N-terminal region of PPR9 fused in-frame to eGFP (N82_PPR9:GFP) construct was introduced into the *P. patens* protoplasts by PEG 4000 transformation method (Schaefer et al. 1991). For transformation, protoplasts were pelleted by centrifugation (10 min, 50 ×g, at 25 °C), and approximately 1.2 × 10^5^ cells were incubated with 30 ~ 50 µg of plasmid DNA suspended in 0.1 M Ca(NO_3_)_2_ within a 25% (w/v) PEG 4000 solution. Transformed cells were imaged 72 h post-transformation using an Olympus FV3000 confocal microscope, and the resulting images were visualized and processed with the Fiji open-source software (Schindelin et al. 2012).

### Mitochondrial transcript analysis in wild type and mutant plants

Total RNA was extracted from approximately 50 mg of plant tissue using RNAzol^®^ RT (R4533, Sigma-Aldrich, Rehovot, Israel) reagent. The isolated RNA was subsequently treated with RNase-free DNase I to remove DNA prior for its use in RT-qPCR analysis. In brief, cDNA was synthesized from 1 to 3 µg of total RNA using SuperScript III First-Strand Synthesis System for RT-PCR (Cat. no. 18080-051, Invitrogen, Rhenium Modi’in, Israel). RNA (11 µl of total RNA in nuclease-free water) was mixed with 1 µl dNTP mix (2.5 mM each nucleotide) and 1 µl random hexamers (1 µg/ml), incubated at 65 °C for 5 min, and immediately chilled on ice for 5 min. A reaction mix containing 4 µl 5× ‘First Strand’ buffer, 1 µl DTT, 1 µl RNasin RNase inhibitor, and 1 µl SuperScript III, for each cDNA synthesis reaction, was prepared on ice, and added to each RNA sample. Samples were then incubated at 25 °C for 5 min for primer annealing, followed by 60 min at 50 °C for cDNA synthesis, and 15 min at 70 °C to inactivate the enzyme. Gene-specific oligonucleotides were designed to amplify both mRNA (‘m’) and pre-mRNA (‘p’) transcripts (Table S4) in qPCR. For normalization, *Arabidopsis thaliana* reference genes, *GAPDH* (*AT1G13440*), *ACTIN2* (*AT3G18780*), *18 S rRNA* (*AT3G41768*) and the mitochondrial *26 S rRNA* (*rrn26*, *ATMG00020*) were used as internal calibrators. Data were normalized to the average expression level of all reference genes measured in each sample.

RNase-protection assays (Cohen et al. 2014; Keren et al. 2009; 2012; Ostersetzer et al. 2005) were performed with in vitro transcribed labeled RNA fragments complementary to exon-exon or exon-intron regions in each transcript. DNA fragments, amplified by PCR using a 5′ primer containing the T7 RNA polymerase promoter sequence fused to the gene of interest, were used to generate RNA probes (labeled with Fluorescein-12-UTP, Cat. 11427857910, Sigma-Aldrich Israel Ltd, Rehovot, Israel) complementary to organellar transcripts, and subjected to RNase T1 digestion. Detection was carried out using the Molecular Dynamics Storm system, and analyzed by the ImageQuant software at the Center for Molecular Dynamics (HUJI, Jerusalem, Israel).

### Enrichment of organellar membranes from wild type and mutant seedlings

Enriched mitochondria membrane fractions from wild type and mutant plants were prepared as previously described (Pineau et al. 2008) with minor adjustments. Approximately 200 mg of whole plant tissue was homogenized in 2.0 mL of membrane extraction buffer (75 mM MOPS-KOH, pH 7.6; 0.6 M sucrose; 4 mM EDTA; 0.2% polyvinylpyrrolidone-40; 8 mM L-cysteine; and 0.2% bovine serum albumin). The homogenate was filtered through a single layer of Miracloth to remove cell debris and centrifuged at 1300 × g to pellet cell fragments and thylakoid membranes. The crude mitochondria-enriched membrane fraction was collected by a sequential centrifugation at 22,000 ×g for 10 min at 4 °C.

### Analysis of the steady state levels of organellar proteins in wild type and mutant plants

Protein was extracted from mitochondria-enriched membrane fractions corresponding to approximately 200 mg of fresh whole-plant tissue, solubilized in protein sample buffer, and separated by SDS-PAGE (Laemmli 1970). For immunoblotting, the proteins were transferred onto a PVDF membrane and incubated overnight at 4 °C with specific antibodies (Table S5). Detection was performed using chemiluminescence following incubation with horseradish peroxidase (HRP)-conjugated secondary antibody.

### Blue Native (BN-) PAGE analysis of mitochondrial respiratory complexes

BN-PAGE of mitochondria-enriched membrane fractions was performed generally according to the method described by Pineau, et al. (Pineau et al. 2008). In brief, mitochondria-enriched organellar membrane fractions, equivalent of 200 mg of ~ 2 month-old liquid MS-grown Arabidopsis seedlings, was solubilized in 200 µL of 1.5% (w/v) n-Dodecyl β-D-maltoside (DDM, CAS no. 69227-93-6) solution containing 0.6% (w/v) Brilliant Blue G (CAS no. 6104-58-1). Insoluble materials were removed by a centrifugation step (i.e., 20,000 xg). Solubilized membrane fractions were loaded onto a native gel. For immunoblotting, the proteins were transferred to a PVDF membrane, and incubated with primary antibodies (Table S5), as indicated in each blot. Detection was carried out by chemiluminescence assay with horseradish peroxidase (HRP)-conjugated secondary antibody.

### Estimation of the relative accumulation of different organellar proteins in wild type (Col-0) and *ppr9* mutant plants

Detection of organellar proteins was performed by immunoblotting assays and using the ImageQuant^TM^ LAS-4000 system (GE Healthcare Life Sciences, Daniel Biotech, Rehovot, Israel) following chemiluminescence assays with horseradish peroxidase (HRP)-conjugated secondary antibodies. The signals were analyzed and quantified using the Fiji open-source software (Schindelin et al. 2012).

### Respiratory activity in Arabidopsis wild type and mutant-lines

Respiratory oxygen consumption was measured in darkness at 22 °C using a Clark-type oxygen electrode (Hansatech Instruments, Norfolk, UK) connected to Oxygraph Plus software (v1.01), as previously described (Kobaivanov et al. 2026). The electrode was calibrated with sodium dithionite to establish the zero-oxygen baseline. Whole plants (~ 2 month-old liquid MS-grown) were cut into small pieces and pre-equilibrated for 15 min in autoclaved tap water. Subsequently, 50 mg of tissue was transferred to the electrode chamber containing 2.0 ml of autoclaved tap water, and oxygen uptake was monitored continuously.

### Data statement

The Arabidopsis Information Resource (TAIR) database provides genetic and molecular biology data for the model plant *Arabidopsis thaliana* (Reiser et al. 2024). The predicted three-dimensional structures of proteins were obtained from and analyzed using the AlphaFold Protein Structure Database, which provides publicly accessible computational models of protein structures (Varadi et al. 2023). Accession numbers: Arabidopsis mitochondrial genome, BK010421.1; PPR9: gene AT1G03560, protein Q9LR67 (PPR9_ARATH). Data supporting the results of this study can be obtained from the corresponding author upon reasonable request.

## Supplementary Information

Below is the link to the electronic supplementary material.

Supplementary Material 1.

## Data Availability

The Arabidopsis Information Resource (TAIR) database provides genetic and molecular biology data for the model plant *Arabidopsis thaliana* [74]. The predicted three-dimensional structures of proteins were obtained from and analyzed using the AlphaFold Protein Structure Database, which provides publicly accessible computational models of protein structures [103]. Accession numbers: Arabidopsis mitochondrial genome, BK010421.1; PPR9: gene AT1G03560, protein Q9LR67 (PPR9_ARATH). Data supporting the results of this study can be obtained from the corresponding author upon reasonable request.
