# Supplementary material for: PPR9 mediates mitochondrial nad transcript maturation required for complex I biogenesis and early plant development in Arabidopsis
Source: Plant Mol Biol. 2026 Oct 21;116(6):105. doi: 10.1007/s11103-026-01769-9 (PMC13687136; doi:10.1007/s11103-026-01769-9)
Supplement: Supplementary file 1 [file 11103_2026_1769_MOESM1_ESM.pdf]

Supplementary Figure legends

Figure S1. PPR9 protein structure and its predicated RNA biding site.

(a) The deduced amino acid sequence of PPR9 (UniProt Q9LR67 · PPR9_ARATH). The predicted mitochondrial targeting sequence (53 amino acid long, mTP) is highlighted in magenta, with the cleavage sites indicated by arrow and line. Regions of low confidence are colored in black. Predicted PPR motifs are coded blue for regions of high confident, whereas domains of low scoring are highlighted in cyan.

(b) Homology modeling of *Arabidopsis thaliana* (Col-0) PPR9. The 3D model of the PPR9 protein structure was obtained from AlphaFold server [3]. The hypothetical 3D surface was visualized with PyMol. The "i" to "iii" panels represent ribbon structures at different angles, whereas "iv" to "vi" indicate the hypothetical surface of PPR9. The color code is red for negative values, white for near zero values and blue for positive values.

Figure S2. Expression patterns of PPR9 across tissues and developmental stages.

Expression levels and patterns of PPR9 were analyzed using the publicly available 'The Arabidopsis Information Resource' website (TAIR; http://www.arabidopsis.org).

Figure S3. Phenotypic analyses of wild type and *ppr9* mutant plants.

(a) Two-month-old wild type (Col-0), heterozygous (Htz; *PPR9/ppr9*) and complemented (*35S::PPR9/ppr9; Comp.*) plants were grown under optimal growth conditions in a controlled growth chamber. Seeds were collected from each genotype, and the resulting progeny were germinated on MS-agar plates and screened to identify wild type, Htz, homozygous (*ppr9*) and complemented seedlings for subsequent analyses of growth, RNA and protein profiles. The lower panel shows representative PCR-based genotyping of progeny derived from heterozygous (Htz; *PPR9/ppr9*) plants transformed with the native PPR9 coding sequence under the control of the CaMV 35S promoter (*35S::PPR9*). Plants homozygous for the T-DNA insertion at the *PPR9* locus and carrying the *35S::PPR9* transgene are marked with red asterisks. The lower right panel shows RT-qPCR analysis of complemented and wild type plants using primers specific to the transgene, confirming transgene expression (at the RNA level) in the Comp. *ppr9* lines.

(b) Developmental phenotypes associated with *in vitro* rescued homozygous *ppr9* mutants. Siliques at the green-mature stage were collected from heterozygous plants. The siliques contained

green seeds (wild type or heterozygous) and translucent, or pale-green seeds, containing homozygous *ppr9* embryos. Green and pale-green seeds, obtained from surface-sterilized siliques, were germinated and grown on MS-agar plates supplemented with sugar and vitamins (see Materials and methods) until they reached the L6 stage [2], and were subsequently transferred to a liquid MS medium for further biomass production [1, 4]. The image shows representative ~2-month-old *in vitro*-germinated wild type (Col-0), heterozygous (Htz) and eight individual homozygous *ppr9* plants (*ppr9* 1-to-8), which were grown in liquid medium. Phenotypic variation among *in vitro* rescued homozygous *ppr9* plantlets is shown. Plantlets 2 to 4 exhibited a bushy morphology, whereas *ppr9* lines #5 and #6, initially grew in liquid culture but later (after several weeks) collapsed. Other plantlets, such as *ppr9* #7 and #8 failed to develop further and died after transfer from MS plates to the liquid media. Genotypes were confirmed by PCR and sequencing. Scale bars represent 1 cm, as indicated.

Figure S4. mt-RNA metabolism defects associated with *ppr9* mutants.
Total RNA was extracted from wild-type (Col-0) and *in vitro* rescued homozygous *ppr9* mutants. The relative (steady-state) accumulation of specific mitochondrial transcripts was evaluated by RNase-protection assays and RT-qPCR analyses, using probes or oligonucleotides (respectively) (Table S4) designed to specific exon-exon or exon-intron regions, with Col-0 RNA used as calibrator. In each case, "m" denotes the mature (mRNA) transcript, whereas "p" denotes the precursor (pre-mRNA) transcript. Mature (m) transcripts were detected using complementary labeled RNA fragments corresponding to coding regions, whereas precursor (p) transcripts were detected using RNA probes spanning exon-intron or intron-exon junctions. The specific exons or introns are indicated between each blot. For analysis of *nad2* intron 2 (i2), we used an RNA probe spanning exon 2 (ex2) and intron 2 (i2). Processing of *nad2* i3 was assayed using a complementary RNA probe spanning ex3 and i3. Similarly, processing of *nad7* i1 and i2 was analyzed using RNA probes spanning exon 1-intron 1 (ex1-i1) and exon 2-intron 2 (ex2-i2), respectively. For *cox2* and *rpl2*, labeled RNA probes spanning the first exon and the adjacent intron were used. The intronless *nad6* transcript was analyzed using an RNA probe complementary to the coding region. The positions of the probes (shown in red) are indicated on the gene diagrams below the blots. The histograms show the relative accumulation (i.e., Log_2 ratios) of mRNA (light blue) and pre-RNA (orange) transcripts in *ppr9* mutants compared with those of germinated embryos of Col-0 plants.

Values represent the means of nine RT-qPCRs corresponding to 3 biological replicates (error bars represent one standard deviation), after normalization to the *GAPDH* (*AT1G13440*), *ACTIN2* (*AT3G18780*), *18S rRNA* (*AT3G41768*), and the mitochondrial *26S rRNA* (*rrn26*, *ATMG00020*) genes. Schematic gene structures are shown below the representative RNA blots, with scale bar represents 500 bp.

Figure S5. Loss of PPR9 impairs mitochondrial complex I biogenesis.

Crude organellar membrane preparations from germinated embryos at the torpedo stage of wild type (Col-0), heterozygous (*HtZ*; *PPR9/ppr9*), complemented (Comp.; *35S::PPR9/ppr9*), and *in vitro* rescued homozygous *ppr9* mutants, were analyzed by BN-PAGE and immunoblotting with antibodies against NAD9 and Rubisco. Arrows indicate native holo-CI (~1,000 kDa) and the native Rubisco holoenzyme (~550 kDa).

References

1. Best C, Mizrahi R, Edris R, Tang H, Zer H, Colas des Francs-Small C, Finkel OM, Zhu H, Small ID, Ostersetzer-Biran O: MSP1 encodes an essential RNA-binding pentatricopeptide repeat factor required for nad1 maturation and complex I biogenesis in Arabidopsis mitochondria. New Phytol 238: 2375-2392 (2023).
2. Boyes DC, Zayed AM, Ascenzi R, McCaskill AJ, Hoffman NE, Davis KR, Görlach J: Growth stage–based phenotypic analysis of Arabidopsis. A model for high throughput functional genomics in plants. The Plant cell 13: 1499-1510 (2001).
3. Jumper J, Evans R, Pritzel A, Green T, Figurnov M, Ronneberger O, Tunyasuvunakool K, Bates R, Žídek A, Potapenko A, Bridgland A, Meyer C, Kohl SAA, Ballard AJ, Cowie A, Romera-Paredes B, Nikolov S, Jain R, Adler J, Back T, Petersen S, Reiman D, Clancy E, Zielinski M, Steinegger M, Pacholska M, Berghammer T, Bodenstein S, Silver D, Vinyals O, Senior AW, Kavukcuoglu K, Kohli P, Hassabis D: Highly accurate protein structure prediction with AlphaFold. Nature 596: 583-589 (2021).
4. Shevtsov-Tal S, Best C, Matan R, Chandran SA, Brown GG, Ostersetzer-Biran O: nMAT3 is an essential maturase splicing factor required for holo-complex I biogenesis and embryo development in *Arabidopsis thaliana* plants. Plant J 106: 1128-1147 (2021).

Table S1. PPR9 mt-RNA gene-loci binding predictions.

a. PPR motif / RNA-base binding prediction.

PPR motif	Amino acids at 5' and 35'	related RNA base
P*	-	-
PPR-1	NT	C>U
PPR-2	ND	U>C>G
PPR-3	ND	U>C>G
PPR-4	MP	N
PPR-5	SN	A
PPR-6	TD	G>>A
PPR-7	SN	A
PPR-8	SD	G>>A>U
PPR-9	NT	C>U
PPR-10	TT	A>G
PPR-11	RD	N
PPR-12	DP	N
PPR-13	TM	N

* - a degenerated domain

b. mt-RNA gene-loci related to the predicted binding site of PPR9.

Target	mtDNA loci		Gene	Feature	Strand
	Start	End			
CTTTAGAATGAAA	96692	96704	*nad2 intron 3 (nad2.2)*	intron	-
TGTAAGAGCCAGG	244582	244570	*nad7 intron 1*	intron	-
CTGGAAATTGAAT	243506	243518	*nad7 intron 2*	intron	-
Additional gene-loci predicted for the PPR9 binding code, ranked according to their matching scores					
CTTGAGAATGAGA	68408	68420	*atp1*	exon	-
TCTGAGAGTGAGA	68021	68033	*atp1*	exon	-
TTGAAAAACAAAT	67288	67300	*atp1*	exon	-
TGGAAAAACGACT	68085	68097	*atp1*	exon	-
TTCTAAAGCGGTA	68655	68667	*atp1 5'UTR*	5'UTR	-
CTCGAAAGCAGAA	362706	362718	*atp4*	exon	+
CTTAAGAATGGTT	362751	362763	*atp4*	exon	+
TTTCAAAGTGACT	362373	362385	*atp4*	exon	+
TTTGAGATTGTCC	49837	49849	*atp6-1*	exon	+
TTTGAGATTGTCC	265198	265210	*atp6-2*	exon	-
TTGAAAAATATCA	265269	265281	*atp6-2*	exon	-
TGCAAAATCAATA	271162	271174	*atp9*	exon	-
CCCGAAAATGCCC	270804	270816	*atp9 3'UTR*	3'UTR	-
TCCAAGATCAAAG	271268	271280	*atp9 5'UTR*	5'UTR	-

TCCGAGAATGTTG	17763	17775	*ccmB 3'UTR*	3'UTR	-
TTCCAGAACAGGA	191629	191641	*ccmFC exon 2*	exon	+
TCTTAAAACAAGG	316335	316347	*ccmFn1*	exon	+
CCGTAGAACGTAT	292086	292098	*ccmFn2*	exon	+
CGGTAAATCGGGG	291544	291556	*ccmFn2 5'UTR*	5'UTR	+
TTTAAAAGTATGT	181907	181919	*cob*	exon	-
CCTTAAATCGTTT	182305	182317	*cob*	exon	-
TTCCAGATTATCC	115009	115021	*cox1*	exon	-
TTTAAGAGTAAGG	8397	8409	*cox2 3'UTR*	3'UTR	+
TTCTAAAATGGTT	6271	6283	*cox2 exon 1*	exon	+
TCCAAGATCGTAC	7463	7475	*cox2 intron*	intron	+
TTTGAGATTGTTC	329737	329749	*cox3 3'UTR*	3'UTR	-
CCCGAAATTAGAT	329759	329771	*cox3 3'UTR*	3'UTR	-
TGGCAGATCATGT	329786	329798	*cox3 3'UTR*	3'UTR	-
TCTCAAAGTAATA	330935	330947	*cox3 5'UTR*	5'UTR	-
TTGTAAATCAAGA	232941	232953	*matR*	exon	+
TGGAAAAGCGTCT	232215	232227	*matR*	exon	+
TCGTAAATTGGTC	232758	232770	*matR*	exon	+
TGGCAAAGTAACT	218912	218924	*mttB*	exon	-
TGCCAAATCGTCG	219068	219080	*mttB*	exon	-
CCCGAAAATGCCC	234186	234198	*nad1 3'UTR (nad1.3)*	3'UTR	+
TTCAAGAATGTCT	83850	83862	*nad1 5'UTR (nad1.1)*	5'UTR	-
TTTTAGAATGGCT	83091	83103	*nad1 exon 1 (nad1.1)*	exon	-
TTCGAAATTGATT	83145	83157	*nad1 exon 1 (nad1.1)*	exon	-
CGCAAAAGCAGAT	59339	59351	*nad1 exon 3 (nad1.2)*	exon	+
TGGGAGAGTATGC	230332	230344	*nad1 exon 4 (nad1.3)*	exon	+
CCGGAAAGTGTTC	234051	234063	*nad1 exon 5 (nad1.3)*	exon	+
TTTGAGAACATAT	82267	82279	*nad1 intron 1.1 (nad1.1)*	intron (trans splicing)	-
CCGTAAATTGAGG	82571	82583	*nad1 intron 1.1 (nad1.1)*	intron (trans splicing)	-
CCTGAAATTACAT	57583	57595	*nad1 intron 1.2 (nad1.2)*	intron (trans splicing)	+
CGTGAGAGCAAGG	58688	58700	*nad1 intron 2 (nad1.2)*	intron	+
TCTAAAAGCGGGA	58826	58838	*nad1 intron 2 (nad1.2)*	intron	+
TCGGAAAGCACGG	59220	59232	*nad1 intron 2 (nad1.2)*	intron	+
TGCGAGAATAACT	58788	58800	*nad1 intron 2 (nad1.2)*	intron	+
TCTCAAAGTAAAG	233459	233471	*nad1 intron 4 (nad1.3)*	intron	+
CGCGAGAGTGTCA	233779	233791	*nad1 intron 4 (nad1.3)*	intron	+
TCTAAGATTAGGG	230547	230559	*nad1 intron 4 (nad1.3)*	intron	+
TTCAAGATCACTG	97894	97906	*nad2 exon 3 (nad2.2)*	exon	-
TTTTAGAATGTTG	163396	163408	*nad2 intron 2.1 (nad2.1)*	intron (trans splicing)	+
CGCGAAATCAGAG	163633	163645	*nad2 intron 2.1 (nad2.1)*	intron (trans splicing)	+
CCGCAAAGCAAAA	97067	97079	*nad2 intron 3 (nad2.2)*	intron	-
CTTAAAATTATGT	95972	95984	*nad2 intron 3 (nad2.2)*	intron	-
TGCTAAAGCACCT	97170	97182	*nad2 intron 3 (nad2.2)*	intron	-
CGGGAAAGCGCTG	95618	95630	*nad2 intron 3 (nad2.2)*	intron	-
TCCTAGATTGAAG	96880	96892	*nad2 intron 3 (nad2.2)*	intron	-

TCGGAGATCGAGA	95830	95842	*nad2 intron 3 (nad2.2)*	intron	-
CCCCAAAGTAGAA	94320	94332	*nad2 intron 4 (nad2.2)*	intron	-
TTCAAGAATACGA	215458	215470	*nad4 exon 1*	exon	-
CCCGAAAGCGTGC	215115	215127	*nad4 exon 1*	exon	-
TGTTAGAACATTT	215549	215561	*nad4 exon 1*	exon	-
CGTCAAAGTGCCT	213404	213416	*nad4 exon 2*	exon	-
CCCCAAAGTGTTC	207637	207649	*nad4 exon 4*	exon	-
TCTCAGAATAAAA	214306	214318	*nad4 intron 1*	intron	-
TTCAAGAATAGGG	214334	214346	*nad4 intron 1*	intron	-
CCGAAAAGTAAAA	213823	213835	*nad4 intron 1*	intron	-
CGCGAGAATGGAT	214754	214766	*nad4 intron 1*	intron	-
TGCTAAAACAAAG	214471	214483	*nad4 intron 1*	intron	-
TCGTAAAGTAGTC	214997	215009	*nad4 intron 1*	intron	-
CGGCAAATCAAAA	214380	214392	*nad4 intron 1*	intron	-
TTGAAGAGTACCT	210323	210335	*nad4 intron 2*	intron	-
TCGGAAAGCACCC	211066	211078	*nad4 intron 2*	intron	-
TTTCAAATCGGAT	212702	212714	*nad4 intron 2*	intron	-
CCGCAGAACGAGA	210953	210965	*nad4 intron 2*	intron	-
CCGTAGATCGGAA	211073	211085	*nad4 intron 2*	intron	-
TCGTAAATCAAAA	212822	212834	*nad4 intron 2*	intron	-
TCGGAGAGCACTT	207733	207745	*nad4 intron 3*	intron	-
TGGCAGAGCACGA	209160	209172	*nad4 intron 3*	intron	-
CTCGAAATTAAAT	234338	234350	*nad5 5'UTR (nad5.1)*	5'UTR	+
TTTCAAAGCATTA	236211	236223	*nad5 exon 2 (nad5.1)*	exon	+
CTTCAAAGTGGAT	27194	27206	*nad5 exon 4 (nad5.3)*	exon	+
TCGGAGAGCACTG	235304	235316	*nad5 intron 1 (nad5.1)*	intron	+
CGGAAGATCAAGT	235255	235267	*nad5 intron 1 (nad5.1)*	intron	+
CTTGAGAATAAAG	360217	360229	*nad5 intron 3.1 (nad5.2)*	intron (trans splicing)	+
CCCAAGAGCGAGG	360248	360260	*nad5 intron 3.1 (nad5.2)*	intron (trans splicing)	+
CTGAAAAGCAGTT	27515	27527	*nad5 intron 4 (nad5.3)*	intron	+
TGCTAGAGCACGG	27918	27930	*nad5 intron 4 (nad5.3)*	intron	+
CGGGAGAATGAAG	27928	27940	*nad5 intron 4 (nad5.3)*	intron	+
TTGTAAAGTAGTA	245496	245508	*nad7 5'UTR*	5'UTR	-
TGGAAAAACAAAA	245564	245576	*nad7 5'UTR*	5'UTR	-
CCCAAGAACACGC	243062	243074	*nad7 exon 3*	exon	-
TTTCAAATCATTC	242960	242972	*nad7 exon 3*	exon	-
TCGTAAAATAAGA	239367	239379	*nad7 exon 5*	exon	-
CCCAAGAACAACC	241920	241932	*nad7 intron 3*	intron	-
TCTGAGAATGATT	241701	241713	*nad7 intron 3*	intron	-
TCCAAGATCAGAA	240889	240901	*nad7 intron 4*	intron	-
TTGGAAAGTACCC	240272	240284	*nad7 intron 4*	intron	-
TGGGAAAACGCTG	240373	240385	*nad7 intron 4*	intron	-
CTCGAAAACGAAG	24861	24873	*nad9*	exon	+
TCTCAAATTGCAT	24796	24808	*nad9*	exon	+
TTGAAGAGCAAGA	24620	24632	*nad9 5'UTR*	5'UTR	+

TCCAAAATCATAA	328398	328410	*ORF100A*	ORF	+
CCTCAGAATAAGC	226458	226470	*ORF102B*	ORF	-
TTCCAGAGTACCT	257724	257736	*ORF106B*	ORF	+
CTTGAAAGCGGTG	309370	309382	*ORF106D*	ORF	+
CTCGAAAGCAAAG	284716	284728	*ORF106E*	ORF	+
TCGGAAAACAAAC	275131	275143	*ORF107G*	ORF	-
TCCCAAAGCACTA	327198	327210	*ORF109B*	ORF	+
TGGTAGAGCAGTT	164997	165009	*ORF110A*	ORF	+
CTGAAAAATGGCT	367643	367655	*ORF110B*	ORF	-
CGGTAGAATGTGG	367513	367525	*ORF110B*	ORF	-
TTCTAAAGTGCGA	70411	70423	*ORF113*	ORF	-
TGGTAGAGTACAA	70383	70395	*ORF113*	ORF	-
TTCTAAAGTACCA	13010	13022	*ORF116*	ORF	-
TTTCAAAGTATAT	290770	290782	*ORF117*	ORF	+
TCTAAAATCAAGA	290792	290804	*ORF117*	ORF	+
TTCGAGATCAAAG	287700	287712	*ORF118*	ORF	+
TTTAAAAATGGGT	327888	327900	*ORF119*	ORF	-
CGGTAGATTATGT	327900	327912	*ORF119*	ORF	-
CTTGAGAACGGAT	249797	249809	*ORF122A*	ORF	+
CGGGAAAGCACTT	90540	90552	*ORF136A*	ORF	+
CTCTAGAACAGTT	91306	91318	*ORF136B*	ORF	+
TTTGAGATTGAGA	91208	91220	*ORF136B*	ORF	+
CCCCAAAATGCCA	438	450	*ORF139A*	ORF	-
CCCCAAAATGCCA	194635	194647	*ORF139B*	ORF	-
CGGCAGAACATGT	246418	246430	*ORF141*	ORF	-
CTCCAAAACAGCC	16531	16543	*ORF143*	ORF	+
CGCGAGAGTAGGG	74119	74131	*ORF145B*	ORF	+
CGGGAAATCGGTT	54393	54405	*ORF152A*	ORF	+
CGGGAAATCGGTT	260642	260654	*ORF152B*	ORF	-
TGCAAGAGTATTC	277075	277087	*ORF159*	ORF	+
TGGAAGAACACCA	225691	225703	*ORF160*	ORF	+
CTGAAGAGCGCTC	311623	311635	*ORF187*	ORF	-
TTTTAAATTATTC	42786	42798	*ORF199*	ORF	-
CCCAAGAGCAGCA	346950	346962	*ORF240A*	ORF	+
TTCGAGAACAAAT	346630	346642	*ORF240A*	ORF	+
TCTAAGATCACTG	346570	346582	*ORF240A*	ORF	+
TTGGAGAGTACAC	346604	346616	*ORF240A*	ORF	+
CCGCAAAACGCCA	321212	321224	*ORF240B*	ORF	+
TGTTAGATTGCAA	320834	320846	*ORF240B*	ORF	+
CCTTAAAGCAGTA	270493	270505	*ORF262*	ORF	-
CTCCAAAACGGAC	359404	359416	*ORF275*	ORF	+
CGGTAAATCAAGT	359466	359478	*ORF275*	ORF	+
TTCTAAAATGGTT	84375	84387	*ORF291*	ORF	-
CTGGAGAACGAGA	69013	69025	*ORF294*	ORF	-
TGCAAAATCAATA	31159	31171	*ORF315*	ORF	+

TCTAAAATCATAG	222753	222765	*rpl2 5'UTR*	5'UTR	-
TCTAAGAGCGCCT	222160	222172	*rpl2 exon 1*	exon	-
TCTCAGATCGCTC	222400	222412	*rpl2 exon 1*	exon	-
CGTTAAAGTGACG	220354	220366	*rpl2 intron*	intron	-
TGGCAGAATGCTT	220632	220644	*rpl2 intron*	intron	-
TGGAAGATCATTT	184883	184895	*rpl5*	exon	-
TGGAAAATTGGCT	185164	185176	*rpl5*	exon	-
CTGAAGAACAAAA	23125	23137	*rps3 exon 2*	exon	+
TTCCAAAATAAGT	22378	22390	*rps3 exon 2*	exon	+
TTCTAAATCACTT	22254	22266	*rps3 exon 2*	exon	+
TGTCAAAGTGTGG	23349	23361	*rps3 exon 2*	exon	+
TGCTAAAATAGTG	23160	23172	*rps3 exon 2*	exon	+
TCGGAGAGCACTT	21725	21737	*rps3 intron*	intron	+
TCTCAAAGTATGG	21622	21634	*rps3 intron*	intron	+
CTGGAAAACACCC	20366	20378	*rps3 intron*	intron	+
CCGAAGATTGAGG	160105	160117	*rps4*	exon	+
CGCGAGAGTGTAG	79360	79372	*rps7 5'UTR*	5'UTR	+
TTGGAGATTGAGG	79213	79225	*rps7 5'UTR*	5'UTR	+
TGCGAAAGCATGG	127454	127466	*rrn18*	rRNA	-
CGGTAAAACGGGG	127686	127698	*rrn18*	rRNA	-
TGCCAAAACGGCT	126757	126769	*rrn18*	rRNA	-
TTTTAGAACAGAA	140863	140875	*rrn26*	rRNA	-
TTCCAAAGTGTTC	143671	143683	*rrn26*	rRNA	-
CGGCAAAATGACC	141947	141959	*rrn26*	rRNA	-
TGGCAAAATACGG	143064	143076	*rrn26*	rRNA	-
CGTGAAAACACCC	126085	126097	*rrn5*	rRNA	-
CTGAAGATTACAG	171591	171603	*trnfM*	tRNA	-
TGGTAGAGCATAG	189302	189314	*trnG*	tRNA	+
TGGTAGAGCATTG	19944	19956	*trnK.1*	tRNA	+
TGGTAGAGCATTG	346470	346482	*trnk.2*	tRNA	+
TGGTAGAGCGGTC	43559	43571	*trnN*	tRNA	+
TGCTAAATCGACA	41519	41531	*trnS.2*	tRNA	+
TGCTAAATCGACA	321900	321912	*trnS.4*	tRNA	-
CGGTAGAACGTGG	298918	298930	*trnW*	tRNA	+

Table S2. Quantification data of protein profiles.

a. Assessment of native organellar complex abundances in *ppr9* mutant lines relative to wild type (Col-0) plants.

**Genetic line**	**holo-CI**		**$CIII_2$**	**CIV**	**CV**		**Rubisco**
	**CA2**	**Nad9**			**AtpA**	**AtpB**	
ppr9	n.d.	n.d.	3.15	5.55	3.53	3.14	0.92
**Comp.**	0.51	0.36	0.47	0.32	0.79	0.46	~1.17

n.d., - non-detected

b. Relative accumulation of mitochondrial proteins in wild type and *ppr9* plants (normalized to beta-tubulin).

	***ppr9*/Col-0, normalized**	**Comp./Col-0, normalized**
**Complex I**		
CA2	0.69	0.77
NAD9	n.q.	n.q.
**Complex III**		
RISP	3.22	1.56
**Complex IV**		
COX2	1.14	1.20
**Complex V (ATP synthase)**		
AtpA	1.26	1.38
**Other organellar proteins**		
VDAC1-5	1.81	1.69
AOX1a	~19.6	1.64
**Loading control**	***ppr9*/Col-0**	**Comp./Col-0**
β-Tubulin*	~0.48	~0.61

n.q., not reliably quantifiable

* - equivalent ratios to wild type plants

Table S3. List of oligonucleotides used for the screening of *ppr9* mutants and for generating the GFP construct in this study.

Gene / Construct	Forward oligo (5'→3')	Reverse oligo (5'→3')
ppr9 gene specific (GS-F+R)	GGCTCTGGCAAAAGATGTGG	AAAGCAGGGCAGACACAGAA
GS-R2		GCGAGCACTTTGAATCATCCG
35S-P	AGGTGGCTCCTACAAATGCC	
LBb1.3 (T-DNA)		ATTTTGCCGATTTCGGAAC
PPR9-GFP *(PPR9 NcoI F+ PPR9 SpeI R)*	CTCACCATGGCCCGAAGATTTTACCGGA AGCCCTT	AGTCACTAGTCGACGGCAGGAGATTA CGAT
Comp.	CTCACCATGGCCCGAAGATTTTACCGGA AGCCCTT	GCTGGTCACCAATTCAAGAGTCAAAA CAAGTTTCAAG

Table S4. List of RT-qPCR oligonucleotides designed to specific different exon-exon (analysis of mRNA and processed transcripts) and intron-exon regions (analysis of pre-RNAs) in Arabidopsis mitochondria.

a. List of oligonucleotides used for the analysis of the mRNA profiles in wild type and mutant plants

gene target	oligo's name	sequence (5'→3')
atp1	*atp1F*	TCACTTCGACACGTCTTTGC
	atp1R	GGAATGGCCTTGAATCTTGA
atp6	*atp6-1F*	TCTTTTGCGAGTCAATGCAC
	atp6-1R	TCTCGCGTATCTCACATTGC
atp8	*atp8F*	CCGTCGACTTATTGGGAAAA
	atp8R	TTCCTTGGCCATGTACAACA
atp9	*atp9F*	CATTCCCTCTGACGTCGAAT
	atp9R	TCGTCGATTCTTACCCTCGT
atp4	*atp4F*	GGATCAGCTTGCGAATTTGT
	atp4R	GCAAATTGCTTCCCCACTAA
ccmb	*ccmBF*	TCTTGGAATCACATCCAGCA
	ccmBR	CGAGACCGAAATTGGAAAAA
ccmc	*ccmCF*	AGCTACGCGCAAATTCTCAT
	ccmCR	GCCGTGGCGATATAAACAAT
ccmfc	*ccmFcF*	CACATGGAGGAGTGTGCATC
	ccmFcR	GTGGGTCCATGTAAATGATCG
ccmfn-1	*ccmFN1F*	AGCTCTTGGCATTGCTTTGT
	ccmFN1R	AGTGCCACAATCCCATTCAT
ccmfn-2	*ccmFN2F*	CGTGTCGTTCGTAATGGAAA
	ccmFN2R	TGATAAGCCCACCAACTTCC
cob	*cobF*	TGCCGGAATGGTATTTCCTA
	cobR	GCCAAAAGCAACCAAAACAT
cox1	*cox1F*	GTAGCTGCGGTGAAGTAGGC
	cox1R	CTGCCTGGATTCGGTATCAT
cox2	*cox2F*	TGATGCTGTACCTGGTCGTT
	cox2R	TGGGGGATTAATTGATTGGA
cox3	*cox3F*	CCGTAACTTGGGCTCATCAT
	cox3R	AAACCATGAAAGCCTGTTGC
mttb	*mttBF*	GGGGTCTTTCTTTGGAAACC
	mttBR	TCTCCCTCATTCCACTCGTC
nad1 exons 1-2	*nad1 1-2F*	GACCAATAGATACTTCATAAGAGACCA
	nad1 1-2R	TTGCCATATCTTCGCTAGGTG
nad1 exons 2-3	*nad1 2-3F*	ATTCAGCTTCCGCTTCTGG
	nad1 2-3R	TCTGCAGCTCAAATGGTCTC
nad1 exons 3-4	*nad1 3-4F*	AAAAGAGCAGACCCCATTGA
	nad1 3-4R	TCCGTTTGATCTCCCAGAAG
nad1 exons 4-5	*nad1 4-5F*	AGCCCGGGATCTTCTTGA
	nad1 4-5R	TCTTCAATGGGGTCTGCTC
nad2 exons 1-2	*nad2 exons 1-2F*	GCGAGCAGAAGCAAGGTTAT
	nad2 exons 1-2R	GGATCCTCCCACACATGTTC
nad2 exons 2-3	*nad2 exons 2-3F*	AAAGGAACTGCAGTGATCTTGA
	nad2 exons 2-3R	AATATTTGATCTTAGGTGCATTTTC
nad2 exons 3-4	*nad2exons 3-4F*	GCGCAATAGAAAGGAATGCT
	nad2exons 3-4R	CTATGGGTCTACTGGAGCTACCC
nad2 exons 4-5	*nad2exons 4-5F*	CAAAGGAGAGGGGTATAGCAA
	nad2exons 4-5R	TATTTGTTCTTCGCCGCTTT
nad3	*nad3F*	CGAATGTGGTTTCGATCCTT
	nad3R	GCACCCCTTTTCCATTCATA
nad4 exons 1-2	*nad4 exons 1-2F*	ATTCTATGTTTTTCCCGAAAGC

	nad4 exons 1-2R	GAAAAACTGATATGCTGCCTTG
nad4 exons 2-3	*nad4 exons 2-3F*	AATACCCATGTTTCCCGAAG
	nad4 exons 2-3R	TGCTACCTCCAATTCCCTGT
nad4exons 3-4	*nad4exons 3-4F*	TTCCTCCATAAATTCTCCGATT
	nad4exons 3-4R	TGAAATTTGCCATGTTGCAC
nad4L	*nad4L-F*	GGGGAATCCTCCTTAATAGACG
	nad4L-R	AACGAAAATGGCTAACCCAATA
nad5 exons 1-2	*nad5 exons 1-2F*	TGGACCAAGCTACTTATGGATG
	nad5 exons 1-2R	CCATGGATCTCATCGGAAAT
nad5 exons 2-3	*nad5 exons 2-3F*	TACCTAAACCAATCATCATATC
	nad5 exons 2-3R	CTGGCTCTCGGGAGTCTCTT
nad5 exons 3-4	*nad5exons 3-4F*	AACTCGGATTCGGCAAGAA
	nad5exons 3-4R	GATATGATGATTGGTTTAGGTA
nad5 exons 4-5	*nad5exons 4-5F*	AACATTGCAAAGGCATAATGA
	nad5exons 4-5R	GTTCCTGCGTTTCGGATATG
nad6	*nad6F*	TATGCCGGAAAGGTACGAAG
	nad6R	GTGAGTGGGTCAGTCGTCCT
nad7 exons 1-2	*nad7 exons 1-2F*	ACCTCAACATCCTGCTGCTC
	nad7 exons 1-2R	AAGGTAAAGCTTGAAGATAAGTTTTGT
nad7 exons 2-3	*nad7 exons 2-3F*	GAGGGACTGAGAAATTAATAGAGTACA
	nad7 exons 2-3R	TGGTACCTCGCAATTCAAAA
nad7 exons 3-4	*nad7exons 3-4F*	ACTGTCACTGCACAGCAAGC
	nad7exons 3-4R	CATTGCACAATGATCCGAAG
nad7 exons 4-5	*nad7exons 4-5F*	GATCAAAGCCGATGATCGTAA
	nad7exons 4-5R	AGGTGCTTCAACTGCGGTAT
nad9	*nad9F*	GGATGACCCTCGAAACCATA
	nad9R	CACGCATTCGTGTACAAACC
rpl2	*rpl2F*	CCGAAGACGGATCAAGGTAA
	rpl2R	CGCAATTCATCACCATTTTG
rpl5	*rpl5F*	AAGGGGTTCGACAGGAAAGT
	rpl5R	CGTATTTCGACCGGAAAATC
rpl16	*rpl16F*	GAGCATTTGCCAAACTCACA
	rpl16R	CGGACACTTTCATCGTGCTA
rps3	*rps3F*	CCGATTTCGGTAAGACTTGG
	rps3R	AGCCGAAGGTGAGTCTCGTA
rps4	*rps4F*	ACCCATCACAGAGATGCACA
	rps4R	TCACACAAACCCTTCGATGA
rps7	*rps7F*	CTCGAACTGAACGCGATGTA
	rps7R	AAGCTGCTTCAAGGATCCAA
rps12	*rps12F*	AGCCAAAGTACGGTTGAGCA
	rps12R	TTTGGGTTTTTCTGCACCAT
matR	*matR-F*	AATTTTTGCGAGAGCTGGAA
	matR-R	TTGAACCCCGTCCTGTAGAC
rrn18	*rrn18F*	CGTCACCTGGGTCAAAAACT
	rrn18R	GCTTGAAAACCGAAGTGAGC
rrn26	*rrn26F*	GACGAGACTTTCGCCTTTTG
	rrn26R	CTTGGAGCGAATTGGATGAT
rrn5	*rrn5F*	CCGACCTCGATATGTGGAATCGTC
	rrn5R	TGGACCATGTCTCCCGAACAATC
18S rRNA (At3g41768)	*18S nucl-F*	AAACGGCTACCACATCCAAG
	18S nucl-R	ACTCGAAAGAGCCCGGTATT
ACTIN2 (At3g18780)	*actin2-F*	GGTAACATTGTGCTCAGTGGTGG
	actin2-R	AACGACCTTAATCTTCATGCTGC
GAPDH (At1g13440)	*GAPDH-F*	TCTCGATCTCAATTTCGCAAAA
	GAPDH-R	CGAAACCGTTGATTCCGATTC
AOX1A	*aox1aF*	AGCATCATGTTCCAACGACGTTTC
	aox1aR	GCTCGACATCCATATCTCCTCTGG
AOX1B	*aox1bF*	GGACCGTGAAATCTCTTCGATGGC

	aox1bR	TCTAGCATCATTGCTCTGCATCCG
AOX1C	*aox1cF*	TCTTCCAGAGGAGGTATGGTTGCC
	aox1cR	AGTGCATAAGCATCCCTCCAACC
AOX1D	*aox1dF*	TTTGCTCGAAGAGGCTGAGAACG
	aox1dR	CTCGTTCGTACCATTTGGGTTGTG
AOX2	*aox2F*	ACGGTGATTCGTGCTGATGAAGC
	aox2R	TCCTTGATTGCGAATGTCAGAAGC
NDA1	*nda1F*	GTATCCAACCGGCGATTTCACG
	nda1R	AGTTACAGTCTCACAATGCACCTC
NDA2	*nda2F*	TGGTGTTGGTCCTTCTCCTTTCG
	nda2R	TCCATTCGTCAATGCCAATCCTTC
NDB1	*ndb1F*	TAACACATTTGGCACTCCTGGTG
	ndb1R	CTCTGTGCATCCTCTACTTCCTTG
NDB2	*ndb2F*	AGGAGGAGAGGTTTCCTCGATTC
	ndb2R	CAGAGCTCGTCTATTACCCTGACC
NDB3	*ndb3F*	TCCAGGAGACTGGGTTTCGATAGG
	ndb3R	CTCTGTGCATCCTCTACTTCCTTG
NDB4	*ndb4F*	TGCTCAAGAGTGCAAATGGTGAGG
	ndb4R	CCTTGTTGTGATGCAACCTGTGC
NDC1	*ndc1F*	CGAGAGCAGTGACAAACAACAGTG
	ndc1R	AATGCACACCCTTGGCCTCTTG

b. List of oligonucleotides designed for the analysis of the 23 organellar group II introns in Arabidopsis.

**Gene**	**Forward oligo (5'→3')**	**Reverse oligo (5'→3')**
rpl2 intron 1 exon 2	TTAGGAAGAGCCGTACGAGG	CGCAATTCATCACCATTTTG
rps3 intron 1 exon 2	AGCCGAAGGTGAGTCTCGTA	TCTACGGCGGGGTCACTAT
cox2 intron 1 exon 2	TGGGGGATTAATTGATTGGA	AGCAGTACGAGCTGAAAGGC
ccmFc intron 1 exon 1	CCCGGATCGAATCAGAGTT	CACATGGAGGAGTGTGCATC
nad1 intron 1 exon 2	GACCAATAGATACTTCATAAGAGACCA	CGTGCTCGTACGGTTCATAG
nad1 intron 2 exon 2	GGTTGGGTTAGGGGAACATC	TCTGCAGCTCAAATGGTCTC
nad1 intron 3 exon 4	AAAAGAGCAGACCCCATTGA	GGGAGCTGTATGAGCGGTAA
nad1 intron 4 exon 5	AGCCCGGGATCTTCTTGA	ACGGAGCTGCATCCCTACT
nad2 intron 1 exon 2	GCGAGCAGAAGCAAGGTTAT	CCCATTCCTAACCAGTGGAG
nad2 intron 2 exon 2	CCCGATCCGATAGTTTACAA	AATATTTGATCTTAGGTGCATTTTC
nad2 intron 3 exon 4	GCGCAATAGAAAGGAATGCT	GGCGAATTTCAAACTTGTGG
nad2 intron 4exon 4	CTTATTCGTGGCAACCTTCC	TATTTGTTCTTCGCCGCTTT
nad4 intron 1 exon 2	CCGTATGATGCGGAAGTCTC	GAAAAACTGATATGCTGCCTTG
nad4 intron 2 exon 3	GCGGAACGACCAGAAAAATA	TGCTACCTCCAATTCCCTGT
nad4 intron 3 exon 4	TCTAGCTTGGTTCGGAGAGC	TGAAATTTGCCATGTTGCAC
nad5 intron 1 exon 2	TGGACCAAGCTACTTATGGATG	TTCGCAAATAGGTCCGACT
nad5 intron 2-exon 2	GTACGATCGTGTCGGGTGA	CTGGCTCTCGGGAGTCTCTT
nad5 intron 3-exon 4	AACTCGGATTCGGCAAGAA	GCCGTGTAATAGGCGACCA
nad5 intron 4 exon 5	AACATTGCAAAGGCATAATGA	CCTGTAAACCCCCATGATGT
nad7 intron 1 exon 2	ACGGTTTTTAGGGGGATCTG	AAGGTAAAGCTTGAAGATAAGTTTTGT
nad7 intron 2 exon 3	AGTGGGAGAGCCGTGTTATG	TGGTACCTCGCAATTCAAAA
nad7 intron 3 exon 4	TAAAGTGAAGTGGTGGGCCT	CATTGCACAATGATCCGAAG
nad7 intron 4 exon 5	CGGCCAAATGACTACAGGAT	AGGTGCTTCAACTGCGGTAT

Table S5. List of antibodies used for the analysis of organellar proteins in wild type and *ppr9* mutant plants.

Antibody	Protein I.D.	origin	serum	dilution	Reference / source
AOX1a	Ubiquinol (Alternative) oxidase 1a	*Arabidopsis thaliana*	Rabbit (polyclonal)	1/1,000	Agrisera antibodies, AS04 054
AtpA	Mitochondrial ATP-synthase subunit 1 (A)	*Nicotiana tabacum*	Mouse (monoclonal)	1/500	Thomas Elton collection
AtpB	Mitochondrial ATP-synthase subunit 2 (B)	*Nicotiana tabacum*	Mouse (monoclonal)	1/500	Thomas Elton collection
CA2	g-carbonic anhydrase-like subunit 2	*Arabidopsis thaliana*	Rabbit (polyclonal)	1/1,000	[3],[4]
COX2	Cytochrome C oxidase subunit-2	*Nicotiana tabacum*	Mouse (monoclonal)	1/100	Thomas Elton collection
NAD9	NADH-dehydrogenase complex subunit-9	*Triticum spp.*	Rabbit (polyclonal)	1/50,000	(Lamattina et al., 1993)
RISP	Rieske iron-sulfur protein	*Arabidopsis thaliana*	Rabbit (polyclonal)	1/5,000	[1]
Porin/VDAC	Voltage-dependent anion-selective channel protein 1-5	*Arabidopsis thaliana*	Rabbit (polyclonal)	1/1,000	Agrisera antibodies, AS07 212
Rubisco	Ribulose-1,5-bisphosphate carboxylase/oxygenase	*Pisum sativum*	Rabbit (polyclonal)	1/10,000	[2]
β-Tubulin	Tubulin beta chain	*Arabidopsis thaliana*	Rabbit (polyclonal)	1/1,000	Agrisera antibodies, AS10 681

1. Carrie C, Giraud E, Duncan O, Xu L, Wang Y, Huang SB, Clifton R, Murcha M, Filipovska A, Rackham O, Vrielink A, Whelan J: Conserved and novel functions for *Arabidopsis thaliana* MIA40 in assembly of proteins in mitochondria and peroxisomes. Journal of Biological Chemistry 285: 36138-36148 (2010).
2. Cohen I, Knopf JA, Irihimovitch V, Shapira M: A Proposed Mechanism for the Inhibitory Effects of Oxidative Stress on Rubisco Assembly and Its Subunit Expression. Plant Physiology 137: 738-746 (2005).
3. Perales M, Eubel H, Heinemeyer J, Colaneri A, Zabaleta E, Braun H-P: Disruption of a nuclear gene encoding a mitochondrial gamma carbonic anhydrase reduces complex I and supercomplex I+III2 levels and alters mitochondrial physiology in Arabidopsis. Journal of Molecular Biology 350: 263-277 (2005).
4. Sunderhaus S, Dudkina NV, Jansch L, Klodmann J, Heinemeyer J, Perales M, Zabaleta E, Boekema EJ, Braun HP: Carbonic anhydrase subunits form a matrix-exposed domain attached to the membrane arm of mitochondrial complex I in plants. J Biol Chem 281: 6482-6488 (2006).

a

predicted cleavage site

MRRFYRKPFSVPLLNRPHCSSQSHCLYKNGDFLSDDSKCSPLSSSRTSVRWVF | NSSSLPPPEWIEPFNDVSDLVKSNRNLLPSPWV
SQILNLLDGSASMESNLDGFCRKFLIKLSPNFVSFVLKSDEIREKPDIAWSFFCWSRKQKKYTHN | **LECYVSLVDVLALAKDVDRIR**
FVSSEIKKFEFPMT | VSAANALIKSFGKLGMVEELLWVWRKMKENGIEPT | LYTYNFLMNGLVSAMFVDSAERVFEVMESGRIKPD |
IVTYNTMIKGYCKAGQTQKAMEKLRDMETRGHEAD | KITYMTMIQACYADSDFGSCVALYQEMDEKGIQVP | PHAFSLVIGGLCKEG
KLNEGYTVFENMIRKGSKPN | VAIYTVLIDGYAKSGSVEDAIRLLHRMIDEGFKPD | VVTYSVVVNGLCKNGRVEEALDYFHTCRFD
GLAIN | SMFYSSLIDGLGKAGRVDEAERLFEEMSEKGCTRD | SYCYNALIDAFTKHRKVDEAIALFKRMEEEEGCDQT | VYTYTILL
SGMFKEHRNEEALKLWDMMIDKGITPT | AACFRALSTGLCLSGKVARACKILDELAPMGVILD | AACEDMINTLCKAGRIKEACKLA
DGITERGREVP | GRIRTVMINALRKVGKADLAMKLMHSKIGIGYERM | GSVKRRVKFTTLLETCFDS

b

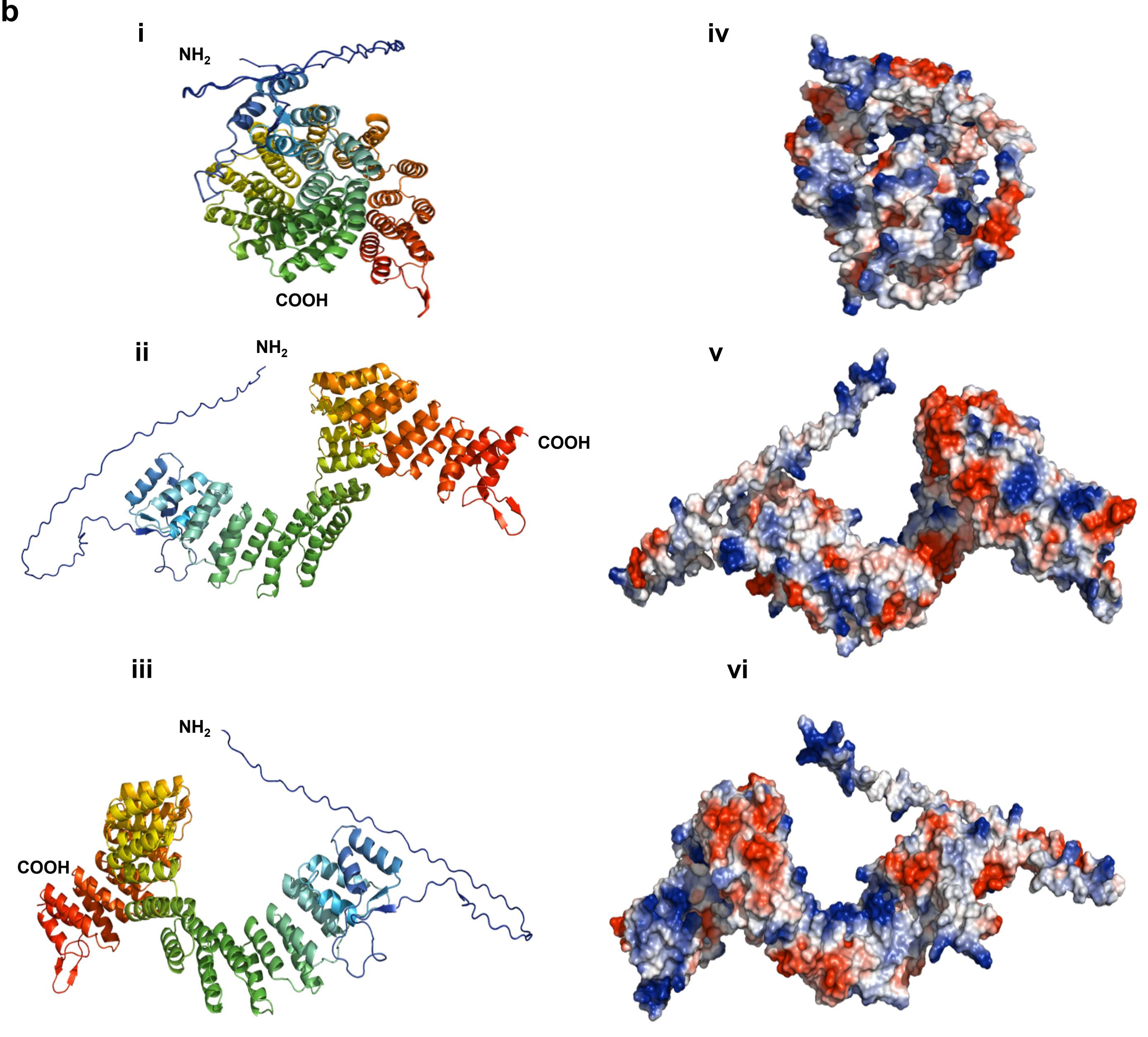

Figure S1

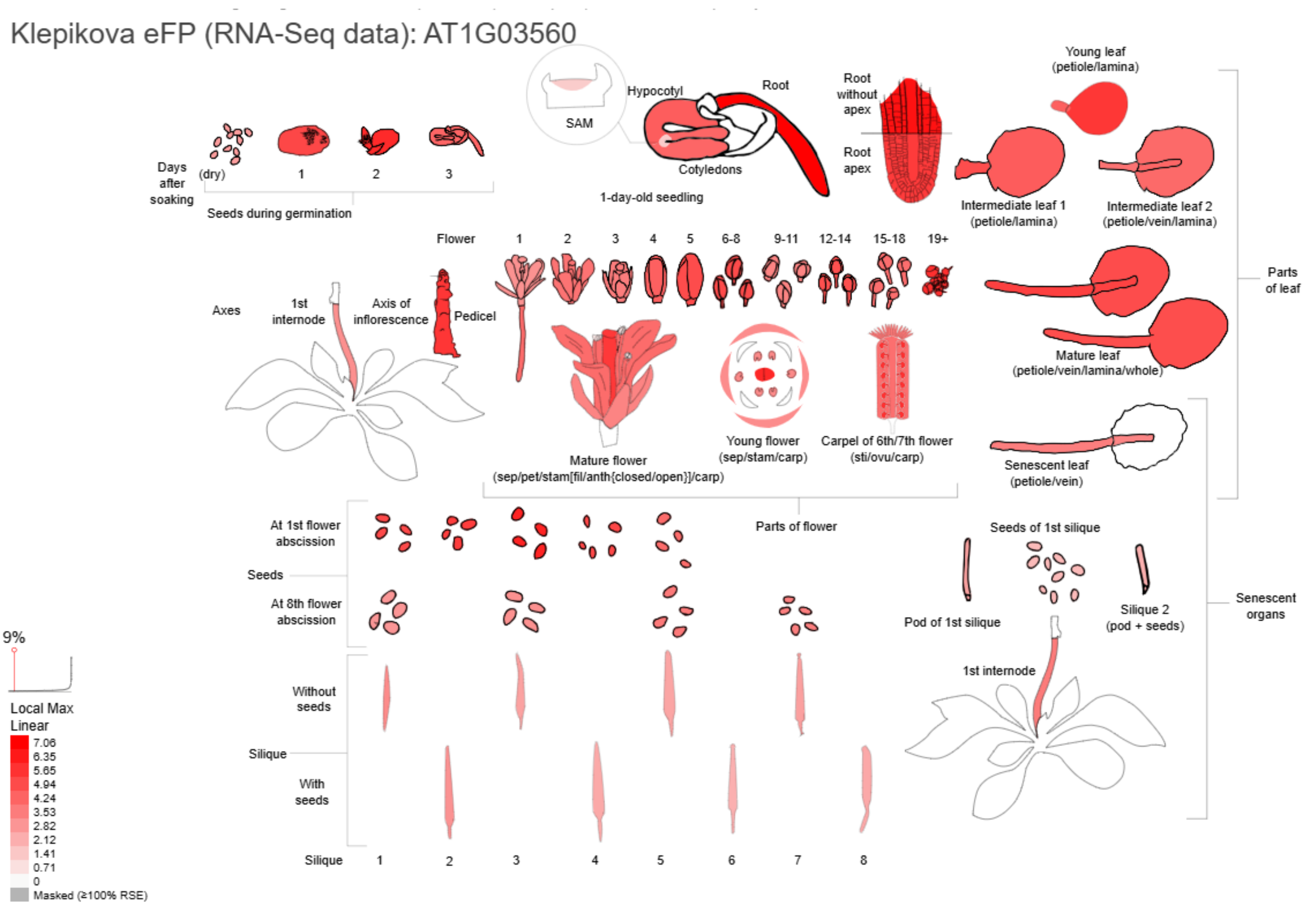

Klepikova eFP (RNA-Seq data): AT1G03560
SAM
Hypocotyl
Root
Root without apex
Root apex
Young leaf (petiole/lamina)
Days after soaking
(dry)
1
2
3
Seeds during germination
Cotyledons
1-day-old seedling
Intermediate leaf 1 (petiole/lamina)
Intermediate leaf 2 (petiole/vein/lamina)
Flower
1
2
3
4
5
6-8
9-11
12-14
15-18
19+
Axes
1st internode
Axis of inflorescence
Pedicel
Parts of leaf
Mature leaf (petiole/vein/lamina/whole)
Mature flower (sep/pet/stam[fil/anth{closed/open}]/carp)
Young flower (sep/stam/carp)
Carpel of 6th/7th flower (sti/ovu/carp)
Senescent leaf (petiole/vein)
At 1st flower abscission
Seeds
At 8th flower abscission
Parts of flower
Seeds of 1st silique
Pod of 1st silique
Silique 2 (pod + seeds)
Senescent organs
1st internode
9%
Local Max
Linear
7.06
6.35
5.65
4.94
4.24
3.53
2.82
2.12
1.41
0.71
0
Masked (≥100% RSE)
Without seeds
Silique
With seeds
Silique
1
2
3
4
5
6
7
8

Figure S2

a ~2-month-old seedlings

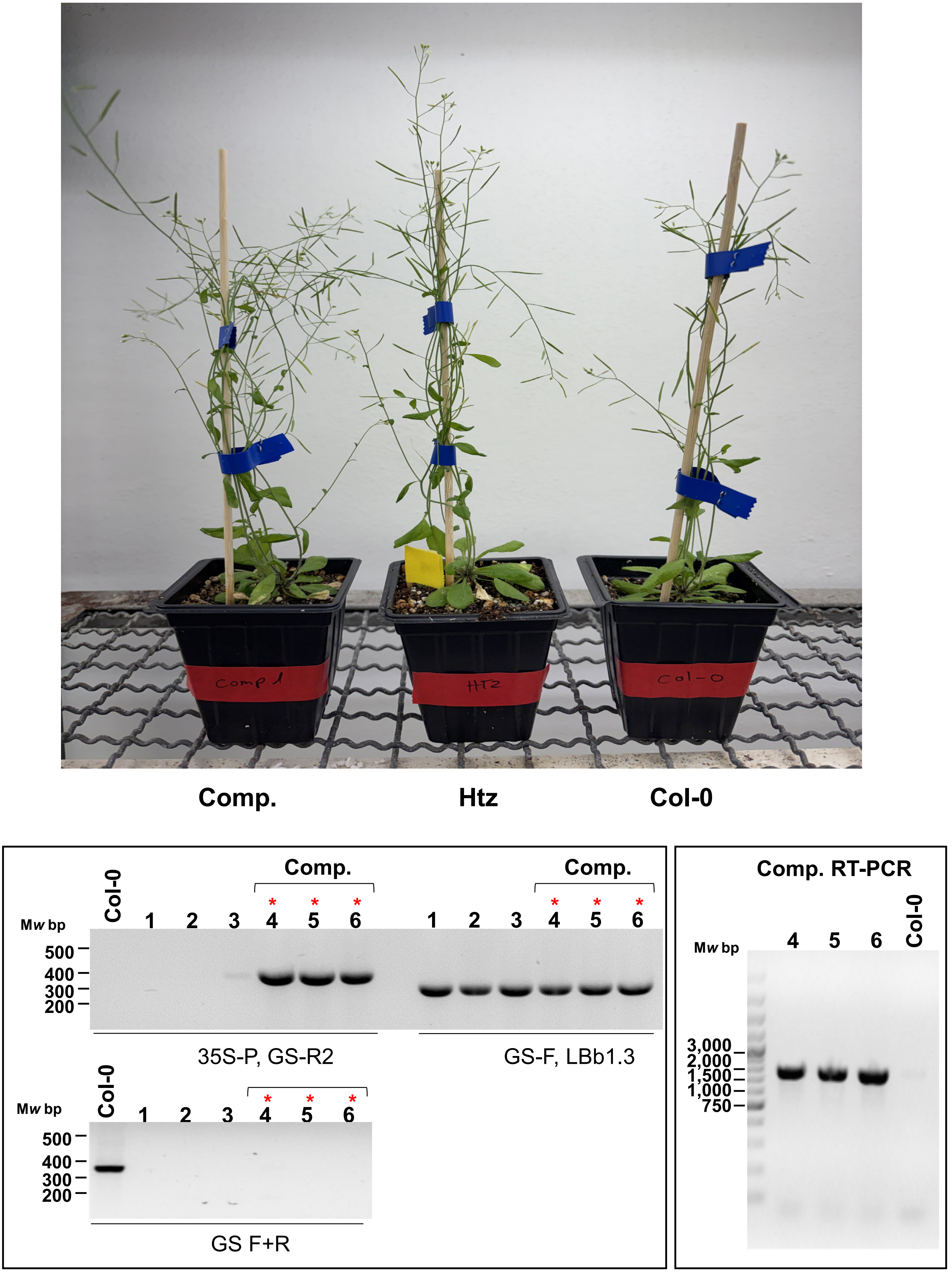

Figure S3a

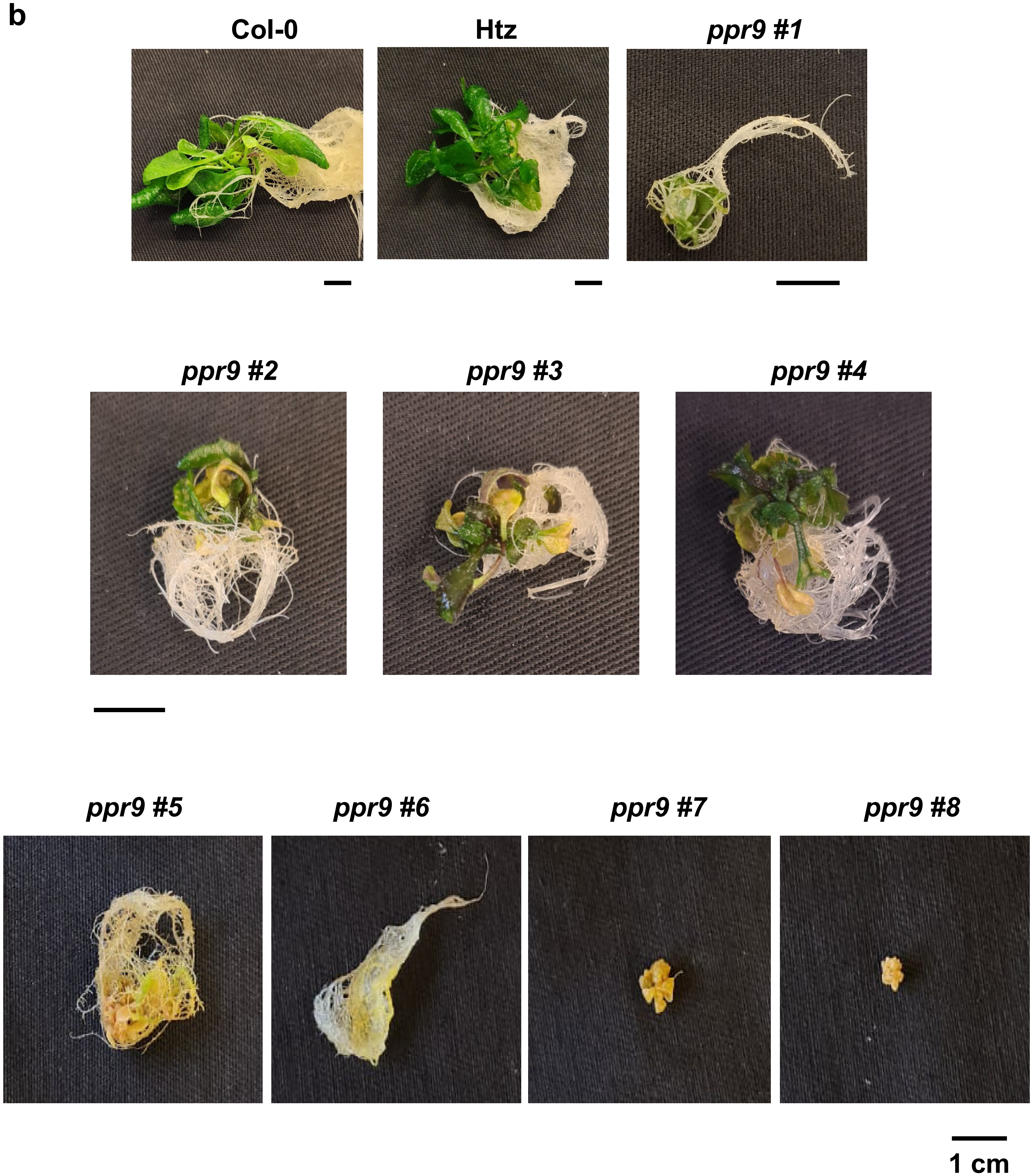
b
Col-0
Htz
ppr9 #1
ppr9 #2
ppr9 #3
ppr9 #4
ppr9 #5
ppr9 #6
ppr9 #7
ppr9 #8
1 cm

Figure S3b

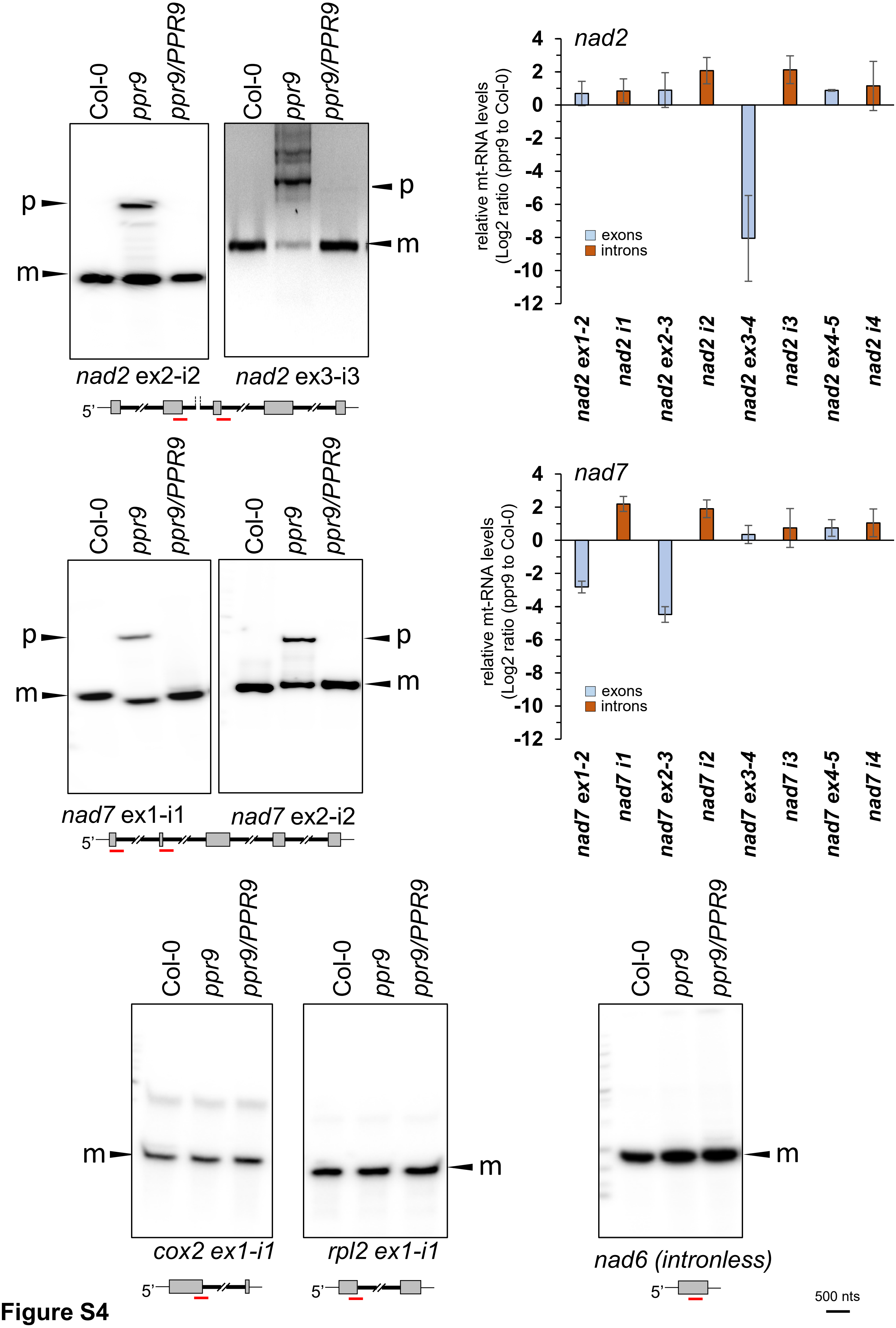
Col-0
ppr9
ppr9/PPR9
p
m
nad2 ex2-i2
nad2 ex3-i3
5'
nad2
relative mt-RNA levels
(Log2 ratio (ppr9 to Col-0))
4
2
0
-2
-4
-6
-8
-10
-12
exons
introns
nad2 ex1-2
nad2 i1
nad2 ex2-3
nad2 i2
nad2 ex3-4
nad2 i3
nad2 ex4-5
nad2 i4
nad7 ex1-i1
nad7 ex2-i2
nad7
nad7 ex1-2
nad7 i1
nad7 ex2-3
nad7 i2
nad7 ex3-4
nad7 i3
nad7 ex4-5
nad7 i4
cox2 ex1-i1
rpl2 ex1-i1
nad6 (intronless)
500 nts

Figure S4

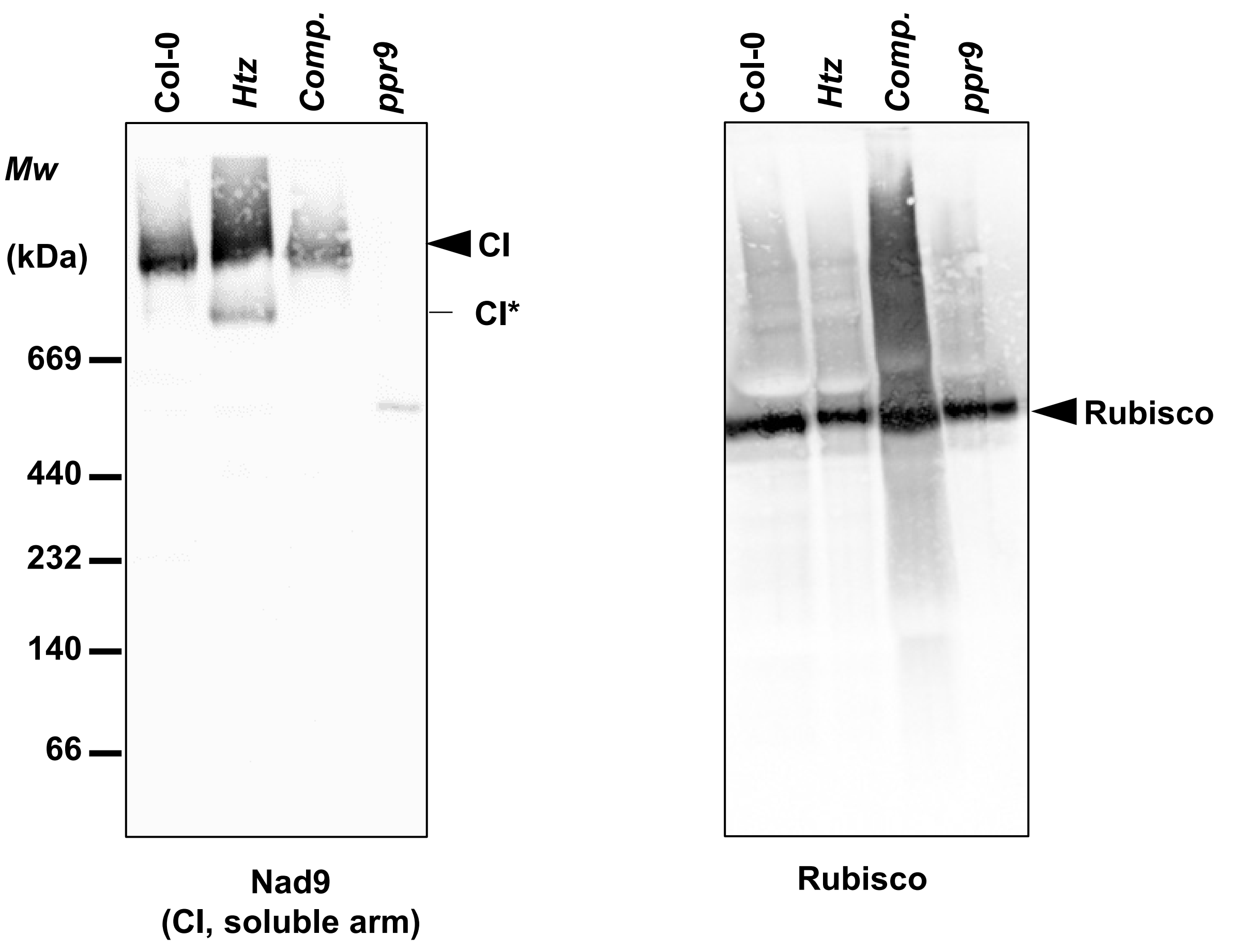
Col-0
Htz
Comp.
ppr9
Mw
(kDa)
669
440
232
140
66
CI
CI*
Nad9
(CI, soluble arm)
Col-0
Htz
Comp.
ppr9
Rubisco
Rubisco

Figure S5
